# Virulence Regulation and Lifestyle Transitions: The Role of c‐di‐GMP and Two‐Component Systems in *Erwinia amylovora* and Their Evolutionary Context Within Enterobacterales

**DOI:** 10.1111/mpp.70228

**Published:** 2026-02-16

**Authors:** Dhirendra Niroula, Kalyani Bhandari, George W. Sundin, Janak R. Joshi

**Affiliations:** ^1^ Plant Sciences and Plant Pathology Department Montana State University Bozeman Montana USA; ^2^ Department of Plant, Soil and Microbial Sciences, Center for Integrated Plant Systems Michigan State University East Lansing Michigan USA

## Abstract

*Erwinia amylovora* , the causative agent of fire blight, poses a threat to pome fruit production worldwide. Disease initiation involves a transition from an epiphytic to an endophytic growth stage, with early activation of the type III secretion system (T3SS) on floral stigmas enabling host tissue invasion. Among the diverse arsenals of *E. amylovora* , the T3SS and its effectors (T3Es), along with the exopolysaccharide amylovoran, represent core pathogenicity determinants. Systemic colonisation and persistence within the host are orchestrated by a complex regulatory network that coordinates transitions between motile planktonic and sessile biofilm‐associated states. Central to this regulation are four conserved two‐component systems (HrpX/HrpY, RcsC/RcsB, GrrS/GrrA and EnvZ/OmpR) and the second messenger cyclic‐di‐GMP (c‐di‐GMP). This review synthesises current knowledge across four interrelated themes: (i) the disease cycle and host colonisation strategies of *E. amylovora* and host defence response, (ii) pathogenicity and virulence factors of *E. amylovora* and their regulatory mechanisms, (iii) regulatory circuits modulating lifestyle transitions during systemic colonisation by the pathogen focusing on the role of c‐di‐GMP and two‐component systems and (iv) evolutionary perspectives on the two‐component systems and c‐di‐GMP along with comparisons to related enterobacterial pathogens. These insights advance our understanding of the molecular basis of fire blight pathogenesis and adaptative strategies employed by *E. amylovora* to thrive in diverse host environments.

## Graphical abstract

*Erwinia amylovora* infects apple blossoms by activating the T3SS, then spreads systemically via amylovoran‐mediated biofilms. Transitions between motile and sessile states are regulated by key two‐component systems and c‐di‐GMP. This review summarises infection biology, virulence factors, regulatory networks and evolutionary insights underlying fire blight pathogenesis.

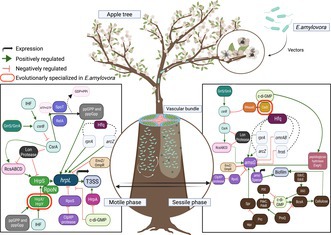

## 1 Background and Introduction

Fire blight, caused by the bacterial pathogen *Erwinia amylovora* , is a highly destructive disease affecting members of the Rosaceae family, including economically important pome fruit crops such as apples (*Malus* spp.) and pears (*Pyrus* spp.). While these pome fruits are among the most severely impacted, the host range of *E. amylovora* extends to several ornamental and wild species, including *Cotoneaster*, *Crataegus* (hawthorn) and *Pyracantha* (Vanneste et al. 1995). However, the introduction of susceptible European pome fruit cultivars by settlers in the 1600s led to widespread outbreaks and the emergence of fire blight epidemics in newly established orchards (Vanneste and Eden‐Green 2000b; van der Zwet and Keil 1979). Since then, the disease has spread globally, causing substantial annual losses in pome fruit production.

*Erwinia amylovora* is a gram‐negative, non‐sporulating, motile bacterium, characterised by the presence of 2–7 peritrichous flagella (Vanneste and Eden‐Green 2000). Notably, *E. amylovora* was the first bacterial species identified as the causal agent of a plant disease in the late 1880s (Burrill et al. 2003). The pathogen can infect various plant tissues, including flowers, shoots, rootstocks and fruits. Among these, floral infections are the most common and economically damaging (Eastgate 2000); *E. amylovora* can also enter plants through natural wounds and openings in shoots and leaves (Millett et al. 2025; van der Zwet et al. 2016). Initial infections typically occur in spring under favourable environmental conditions: temperatures above 18°C, relative humidity above 60% and the presence of rain or wind (Santander and Biosca 2017). The disease spreads rapidly within and between orchards during early summer, particularly when host tissues are actively growing (Luepschen et al. 1961). Characteristic symptoms include water‐soaked lesions, wilting, necrosis resembling fire damage, the distinctive ‘shepherd's crook’ curvature of infected shoots, and the exudation of bacterial ooze. The bacterial pathogenicity factors exopolysaccharide amylovoran, type III secretion system (T3SS) and T3SS effector DspE are the major contributors for the initiation and causation of fire blight symptoms (Kharadi, Selbmann, and Sundin 2021; Yuan, McGhee, et al. 2021). The pathogen has two growth phases, epiphytic and endophytic. The priming of *E. amylovora* pathogen with the T3SS, especially during epiphytic growth on flower stigmas, helps subsequent endophytic colonisation of stem tissues (Cui et al. 2021). The effector DspA/E causes programmed cell death of plant cells while helping systemic spread of the pathogen (Nomura et al. 2023). Exopolysaccharide plays a major role during the endophytic colonisation of bacteria as it is a major contributor for biofilm formation inside the vascular system (Koczan et al. 2009).

Despite extensive research on this historically significant pathogen, many aspects of its biology and management remain unresolved. We are interested in *E. amylovora* virulence mechanisms, how the varied virulence mechanisms are regulated and how these regulatory systems evolved and diverged from related pathogens in the Order Enterobacterales. This review is structured into four main sections. The first part explores the disease cycle of fire blight and the molecular mechanisms underlying pathogen–host interactions. The second section delves into the important factors contributing to *E. amylovora* pathogenesis and virulence. The third part explores the status of two‐component and cyclic di‐GMP (c‐di‐GMP) regulatory systems in *E. amylovora* , and the fourth section discusses evolutionary perspectives of two‐component systems and c‐di‐GMP, particularly in relation to other important genera in the Enterobacterales, with an assessment of how these regulatory systems have contributed to the divergence of this species as a fire blight pathogen.

## 2 Invasion, Colonisation and Systemic Spread

**FIGURE 1:**
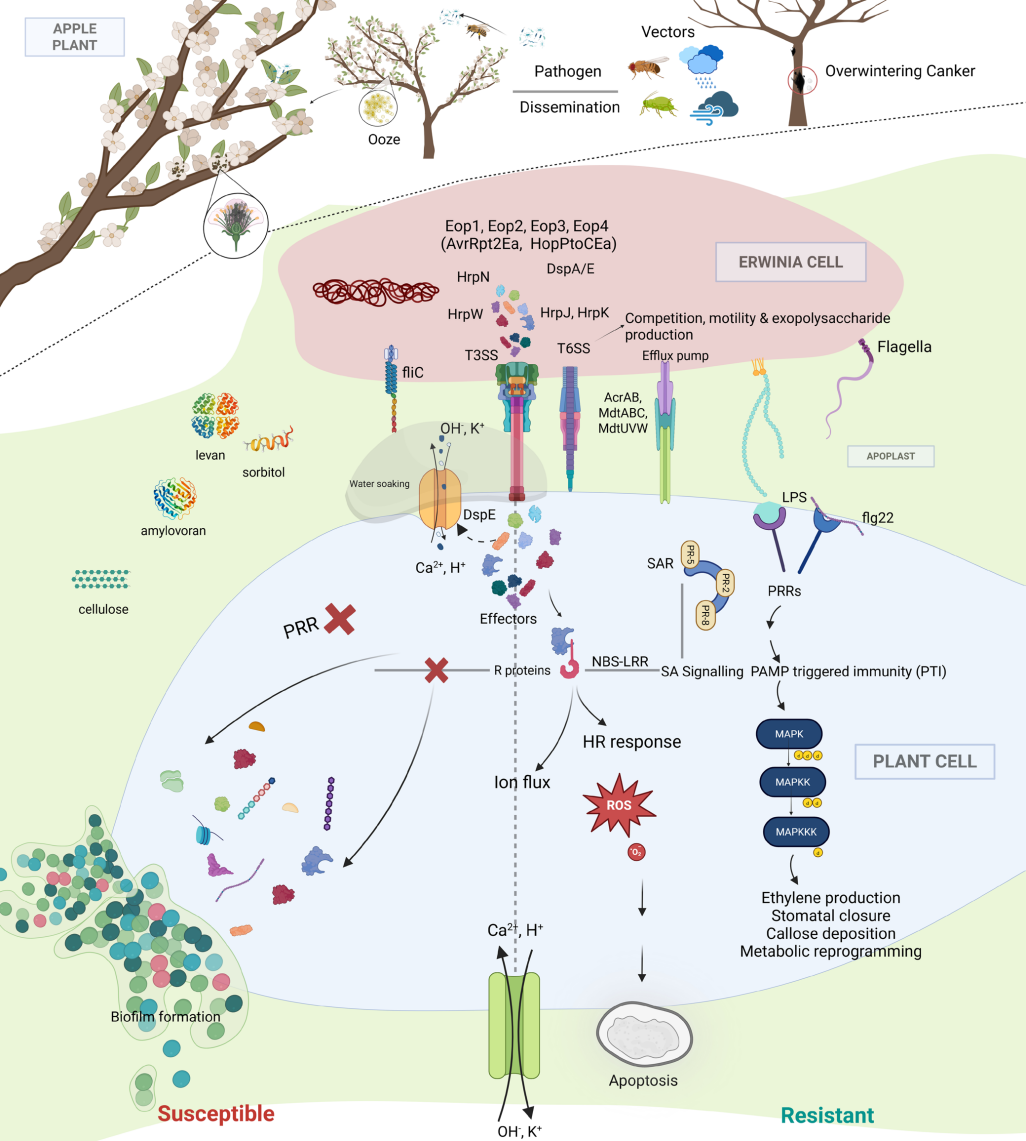
Disease cycle and infection strategy of *Erwinia amylovora* causing fire blight disease. *E. amylovora* overwinters in cankers and emerges as ooze droplets in spring containing bacterial cells that are disseminated via rain, wind and insect vectors, spreading the cells to new tissues. Flower stigmas are the primary location for epiphytic growth of the pathogen; motile *E. amylovora* cells then travel by swimming motility to the nectarthodes. The pathogen colonises primarily using the type III secretion system (T3SS), which delivers effectors (e.g., DspA/E, Eop1, Eop2, Eop3, Eop4, AvrRpt2 and HopPtoCEa) to disrupt host defences, alter ion flux, and cause watersoaking. Exopolysaccharides amylovoran, levan and cellulose also impact virulence and systemic movement via sliding motility. Host plants respond through pattern‐triggered immunity (PTI), recognising PAMPs like flg22 and lipopolysaccharide (LPS), and effector‐triggered immunity (ETI), mediated by NBS‐LRR proteins, activating MAPK cascades, salicylic acid signalling, reactive oxygen species (ROS) bursts and hypersensitive response (HR). However, *E. amylovora* cells survive the host defence response and continue to invade the host systemically. During branch (shoot) infection, *E. amylovora* cells also transition to biofilm formation in xylem vessels in leaves at shoot tips that presumably increases population size before cells emerge from xylem and continue to move systemically downward in infected trees through the apoplast. In susceptible hosts, pathogen colonisation leads to systemic infection and necrosis, whereas resistant plants mount strong PTI/ETI responses that restrict disease.

The disease cycle of *E. amylovora* is initiated by infection from overwintering inoculum sources, including cankers on perennial tissues, mummified fruits and asymptomatic plant parts (Crepel and Maes 2000; de la Peña‐Baca et al. 2023; Weißhaupt et al. 2015). Among these, cankers are most important, serving as primary reservoirs of the pathogen during dormancy and commonly forming on branches, trunks, rootstock and occasionally roots (Santander et al. 2022; van der Zwet et al. 2016) (Figure 1). In response to pathogen detection or attempted invasion, the host plant activates defence mechanisms, including the differentiation of cortical parenchyma cells into a suberised periderm that restricts pathogen movement into adjacent tissues (DebRoy et al. 2004; Thomson 2000).

Among routes of infection, blossom blight is the most economically significant (Wilson et al. 1989). The flower stigma is consistently identified as the primary site of epiphytic colonisation. Once on the stigma, under favourable conditions (optimal temperature, humidity and nutrient availability), *E. amylovora* cells multiply rapidly, reaching populations of 10^6^–10^7^ cells per flower within 1–2 days (Johnson et al. 2006; Pusey and Curry 2004; Slack et al. 2022). Surprisingly, the T3SS is important during the epiphytic growth phase on the stigma, as mutants lacking the T3SS show reduced multiplication on the stigma (Cui et al. 2021; Johnson et al. 2009). In the presence of moisture (e.g., rain or dew), pathogen cells use flagellar motility to migrate to the hypanthium and enter flowers through nectarthodes, the natural opening at the base of the nectary (Bubán and Orosz‐Kovács 2003). The regulation of flagellar motility in *E. amylovora* is complex and is discussed in detail in the pathogenicity and virulence factors section. Pathogen cells also exhibit chemotaxis towards organic acids and aspartate in nectar, but not towards common sugars such as sucrose, glucose or fructose (Raymundo and Ries 1980).

Following entry via the flower nectarthodes, *E. amylovora* establishes infections leading to blossom blight symptoms. Blossom blight is characterised by necrosis of floral tissue, systemic spread of pathogen cells and ooze emergence, which provides two routes of spread that can result in further infection of shoots. Systemic spread within the apoplastic space proceeds downward through the flower pedicel from which *E. amylovora* cells enter shoot tissues. Alternatively, direct infection of shoots can occur through mechanical injuries caused by wind‐blown debris, hail, insect feeding or wounds resulting from the abscission of trichomes, with cells from bacterial ooze serving as the primary inoculum (Millett et al. 2025; Pedroncelli and Puopolo 2023). These infections are marked by characteristic ‘shepherd's crook’ symptoms in young shoots (Suhayda and Goodman 1981a). Remarkably, as few as 100 bacterial cells can initiate shoot blight infection (Crosse et al. 1972). In infected cultivar Gala apple shoots, *E. amylovora* populations can exceed 10^9^ CFU/g and remain elevated for over 20 days, spreading systemically at an average rate of 4.2 cm per day (Dougherty et al. 2025). Microscopy analyses have revealed extensive colonisation of the cortical parenchyma (Dougherty et al. 2025; Schouten 2019), positioning the pathogen near the surface and facilitating ooze production and emergence (Slack et al. 2017), critical for both disease spread and canker formation.

**FIGURE 2:**
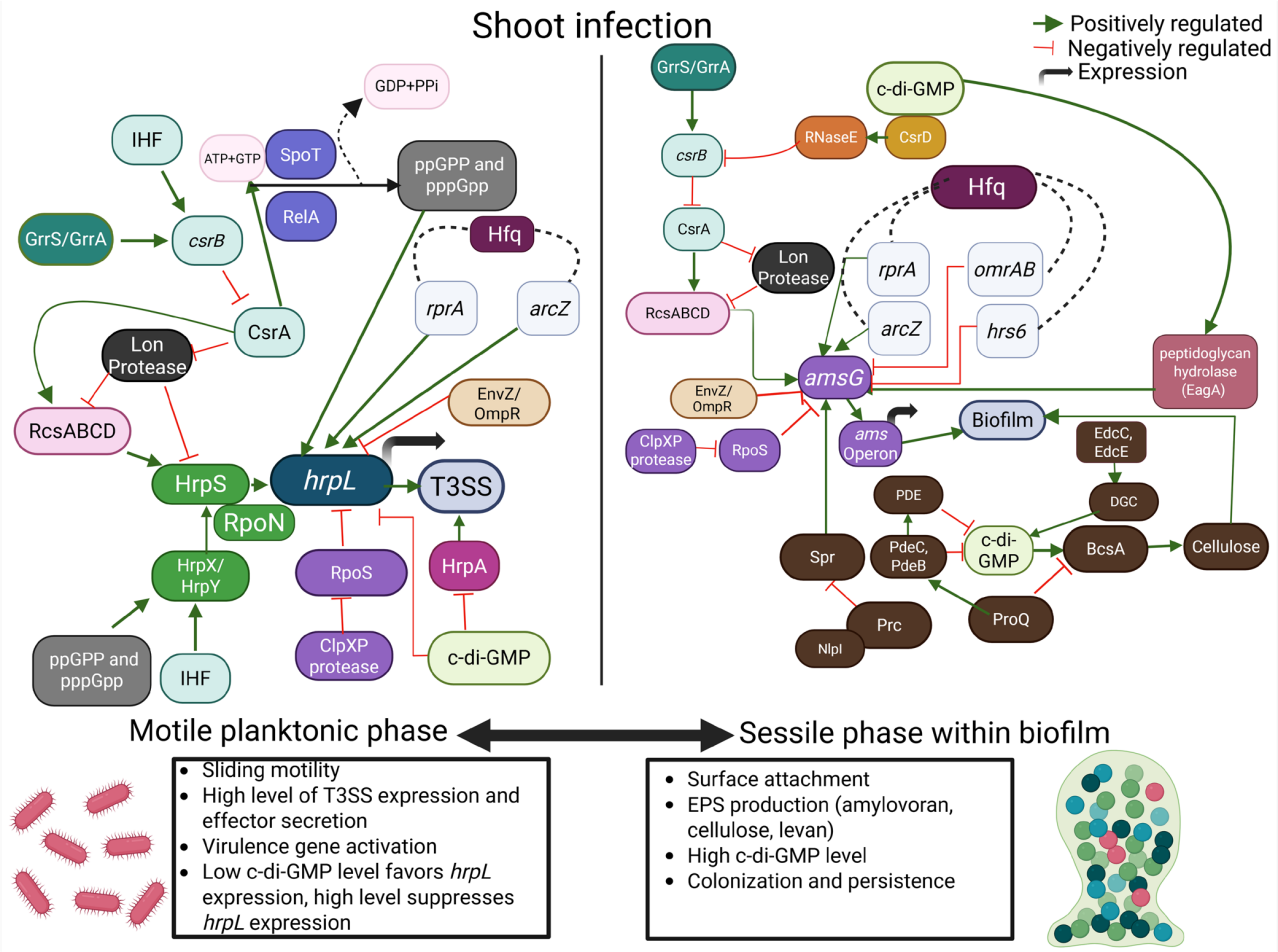
Shoot infection by *Erwinia amylovora* involves a transition between a motile planktonic phase and a sessile biofilm‐associated phase, coordinated by complex regulatory networks. Left panel (motile planktonic phase): Under nutrient limitation, the stringent response triggers RelA/SpoT‐dependent (p)ppGpp accumulation, enhancing RpoN‐dependent transcription of *hrpS*. HrpS, activated by the HrpX/HrpY TCS and IHF, induces *hrpL*, whose product drives expression of type III secretion system (T3SS) structural and effector genes via *hrp*‐box promoters. T3SS output is further shaped by CsrA (antagonised by *csrB*, itself regulated by GrrS/GrrA and IHF), Hfq‐dependent sRNAs (*arcZ*, *rprA*), the RcsBCD and EnvZ/OmpR systems, YhbH and proteolysis mediated by Lon and ClpXP through RpoS turnover. Low intracellular c‐di‐GMP supports *hrpL*/*hrpA* expression, motility and virulence. Right panel (sessile biofilm phase): Elevated c‐di‐GMP suppresses T3SS and motility while promoting extracellular polysaccharide (EPS) production and biofilm formation. Diguanylate cyclases and phosphodiesterases modulate c‐di‐GMP levels, which regulate *amsG* and the *ams* operon through CsrD–RNaseE‐mediated control of *csrB*, CsrA activity and the RcsBCD phosphorelay. Hfq‐associated small RNAs (*arcZ*, *rprA* as positive regulators; *omrAB*, *hrs6* as negative regulators) and ProQ further influence amylovoran and cellulose synthesis. ProQ represses EPS and c‐di‐GMP accumulation by modulating *prc* expression; reduced Prc alters the Prc–NlpI protease complex, which inhibits amylovoran, while the Prc‐degraded hydrolase Spr enhances EPS synthesis. Lon and ClpXP fine‐tune EPS production via turnover of RcsABCD and RpoS. Cellulose synthesis is stimulated by c‐di‐GMP through allosteric activation of BcsA and requires BcsZ for efficient production. Biofilm maturation is reinforced by the peptidoglycan hydrolase EagA, whereas EnvZ/OmpR negatively regulates *amsG*.

Within the apoplast, *E. amylovora* employs the T3SS to inject effector proteins (T3Es), a central virulence mechanism (Jin et al. 2001; Kharadi, Schachterle, et al. 2021; Vrancken et al. 2013). The T3SS is encoded by hypersensitive response and pathogenicity (*hrp*) loci, which are induced by mannitol, salts and ammonium sulphate (Wei et al. 1992). The induction is further promoted by the bacterial alarmone ppGpp, which activates the system under nutrient‐limited conditions (shown in Figure 2) (Ancona et al. 2015; Yang et al. 2020). Transcriptomic analyses show that T3SS genes and their effectors are strongly upregulated within 24 h after inoculation (Puławska et al. 2017). The expression of T3SS genes is tightly regulated by a complex regulatory circuit that is explained in detail in the pathogenicity and virulence factors section.

During the initial phase of shoot blight infection, *E. amylovora* cells invade xylem vessels within leaf veins, forming biofilms. *E. amylovora* produces three key extracellular polysaccharides (EPS: amylovoran, levan and cellulose) that are essential for biofilm formation and virulence (Kharadi, Schachterle, et al. 2021; Oh and Beer 2005). These biofilms protect the pathogen from host defences and sap flow, while supporting population expansion. Entry into xylem vessels may occur directly through wounds or more commonly via parenchyma, guided by EPS production and osmotic or nutrient gradients (Suhayda and Goodman 1981a, 1981b). Biofilm formation is dependent on the second messenger compound c‐di‐GMP, as an *E. amylovora* c‐di‐GMP null mutant is incapable of attachment (Kharadi et al. 2022), a trait that is required for biofilm formation. The regulation of biofilm formation in *E. amylovora* has been discussed in detail in the pathogenicity and virulence factor section below. Further systemic spread of *E. amylovora* in branches also depends on biofilm dispersal, which involves direct rupturing and release of cells from xylem vessels (Geider 2000). Cells released from xylem vessels re‐enter the cortical parenchyma and reinitiate T3SS‐mediated infection (Bogs et al. 1998). The Hfq‐dependent sRNA RprA is a key regulator of biofilm dispersal (Peng et al. 2021). The transition from biofilm to T3SS‐mediated infection is also controlled by an adjustment of intracellular levels of c‐di‐GMP from high to low. Higher levels promote biofilm formation and repress T3SS expression, and lower levels promote T3SS expression and repress biofilm formation (Kharadi et al. 2019). The intracellular levels of c‐di‐GMP in bacterial cells are controlled by a system of diguanylate cyclase and phosphodiesterase enzymes that respond to environmental conditions and synthesise or degrade c‐di‐GMP, respectively (Kharadi, Selbmann, and Sundin 2021, 2022).

## 3 Host Defence Response

The fire blight pathogen has been extensively studied with respect to its disease cycle, epidemiology, pathogenicity and virulence regulation. However, the genetic and biochemical mechanisms underlying host resistance mechanisms remain comparatively underexplored (Kamber et al. 2016). Infection of apple flowers and shoots triggers a multilayered immune response that can be broadly divided into pathogen‐triggered immunity (PTI) and effector‐triggered immunity (ETI) (Figure 1). PTI is initiated when host pattern‐recognition receptors (PRR) detect conserved pathogen‐associated molecular patterns (PAMPs) such as lipopolysaccharide components (lipid A) or the flagellin‐derived peptide Flg22. This recognition activates early defence outputs, including apoplastic alkalinisation, oxidative burst and the accumulation of reactive oxygen species (ROS) (Couto and Zipfel 2016). In apple, *E. amylovora* *flg22* mutants fail to induce callose deposition, and subsequent work demonstrated that the harpin effectors HrpN and HrpW are specifically required for this PTI‐associated response (Boureau et al. 2011).

ETI represents a second, more robust layer of defence activated when intracellular resistance (R) proteins recognise pathogen effectors delivered via the T3SS. Effector recognition typically triggers strong ROS production, localised hypersensitive response (HR), programmed cell death and callose deposition to restrict bacterial spread (Toruño et al. 2016). Among *E. amylovora* effectors, HrpN is notable for its pleiotropic activity like eliciting HR, callose elicitation, activating salicylic acid‐dependent signalling pathways, ultimately contributing to defence responses like systemic acquired resistance (SAR) (Dong et al. 1999; Boureau et al. 2011). Resistance to fire blight in Rosaceae hosts was first reported to be polygenic (Aldwinckle and van der Zwet 1979; Korban et al. 1988), although T3E:R gene‐for‐gene resistance interactions have been identified and characterised in the 2010s, and numerous quantitative trait loci (QTLs) have been identified (Emeriewen et al. 2019; Zeng et al. 2024). However, detailed discussion of these loci is beyond the scope of this review.

## 4 Pathogenicity and Virulence Factors of *E. amylovora*

*Erwinia amylovora* deploys a suite of pathogenicity and virulence factors to establish infection and cause fire blight disease in susceptible hosts. The three established primary pathogenicity determinants are the T3SS, the T3E DspA/E and the EPS amylovoran (Venisse et al. 2001). These are essential for initiating disease. In contrast, virulence factors such as pathogen motility, biofilm formation, siderophore production (e.g., desferrioxamine, DFO), catalases (CatA and CatG) and metabolic pathways for sorbitol, sucrose and sulphur contribute to disease progression but are not sufficient on their own to initiate infection (Piqué et al. 2015). Additional contributors include the Ttype VI secretion system (T6SS) (Smits et al. 2010). This section focuses on the molecular mechanisms and regulatory networks underpinning these factors, beginning with the T3SS.

### 4.1 Type III Secretion System (T3SS)

The T3SS is a critical virulence apparatus that enables *E. amylovora* to inject effector proteins directly into host cells. It is primarily composed of Hrp (hypersensitive response and pathogenicity) and Hrc (HR and conserved) proteins, encoded by genes clustered within a pathogenicity island. This island comprises four regions: the Hrp/Hrc cluster, the HEE (Hrc effectors and elicitors), the HAE (Hrp‐associated enzymes) and the IT (island transfer) region (Oh et al. 2005). Through the hrp‐T3SS, *E. amylovora* delivers a repertoire of effectors and associated proteins into the host apoplast and cytoplasm. These include effectors—DspA/E, Eop1, Eop2, Eop3, Eop4 (AvrRpt2Ea) and HopPtoC_Ea_; harpins—HrpN and HrpW; and other proteins—HrpJ and HrpK (Yuan, Hulin, and Sundin 2021). Many of these effectors have homologues in other phytopathogens such as *Pectobacterium carotovorum, Pseudomonas syringae, Xanthomonas* spp., *Ralstonia* spp. and *Rhizobium* spp. (Joshi et al. 2026; Raymundo 1980).

DspA/E is the central pathogenicity effector in *E. amylovora* , indispensable for disease initiation (Gaudriault et al. 2002; Oh et al. 2005). It interacts with host receptor kinases and preferredoxins to manipulate host cellular processes (Meng et al. 2006). The delivery of DspA/E into host cells is mediated by three chaperones, DspB/F, Esc1 and Esc3, that cooperatively protect it from cytoplasmic degradation and orchestrate secretion and translocation (Castiblanco et al. 2018; Gaudriault et al. 2002). Recent studies have uncovered additional roles for DspA/E, including the formation of a β‐barrel structure that resembles bacterial porins allowing small molecules to move across that induce apoplastic flooding (Meng et al. 2006; Nomura et al. 2023). Additionally, this effector was shown to increase in abscisic acid levels, leading to stomatal closure (Joshi et al. 2026; Pester et al. 2012). Together, these effects enhance water and nutrient availability for bacteria in host tissues, creating a favourable environment for pathogen colonisation and systemic spread. Another effector Eop1, a member of the YopJ/AvrRxv family, exhibits both cysteine protease and acetyltransferase activities (Hotson and Mudgett 2004; Lewis et al. 2011). Eop3, homologous to HopX/AvrPphE, also functions as a cysteine protease, with its N‐terminal domain essential for enzymatic activity (Bocsanczy et al. 2012). Eop4, another cysteine protease, is homologous to the *P. syringae* effector AvrRpt2 and contributes to virulence, as mutants lacking Eop4 show reduced symptoms in immature pear assays (Zhao et al. 2006). Interestingly, expression of *eop4* in transgenic, micrografted cultivar Pinova apple plants was sufficient to cause typical fire blight shepherd's crook symptoms (Schröpfer et al. 2018). The roles of Eop2 and HrpK remains less defined, though they are conserved across related pathogens (Kim and Beer 1998). HrpN is essential for full virulence and facilitates DspA/E translocation, while HrpW elicits an HR in resistant hosts (Choi et al. 2013; Gaudriault et al. 2002). Both harpins are involved in effector delivery.

Regulation of the T3SS in *E. amylovora* is governed by a multilayered network that integrates environmental sensing, transcriptional control and post‐transcriptional modulation (Figure 2). Under nutrient‐limited conditions, activation of the stringent response leads to accumulation of the alarmone (p)ppGpp, which redirects RNA polymerase activity towards the alternative sigma factor RpoN. This shift enhances RpoN‐dependent transcription of *hrpS*. Expression of *hrpS* is further stimulated by the HrpX/HrpY two‐component system, whose activity is positively influenced by Integration Host Factor (IHF) (Lee and Zhao 2018; Yang et al. 2023; Zhao et al. 2009). HrpS, an AAA+ enhancer‐binding protein, cooperates with IHF‐mediated DNA bending to activate transcription of *hrpL*, which encodes the extracytoplasmic function (ECF) sigma factor that serves as the master regulator of the T3SS (Lee et al. 2016). HrpL subsequently drives expression of T3SS structural and effector genes by recognising the conserved ~35‐bp *hrp‐*box motif in their promoters (Wei and Beer 1995). Consistent with this regulatory cascade, mutants defective in (p)ppGpp synthesis exhibit reduced growth, attenuated virulence and strong downregulation of T3SS genes, underscoring the importance of the stringent response in linking stress adaptation to pathogenicity (Ancona et al. 2015; Yang et al. 2020).

Post‐transcriptional regulation also plays a critical role. The global RNA‐binding protein CsrA promotes translation of *hrpL* and downstream T3SS‐associated transcripts (Ancona et al. 2016). CsrA activity is antagonised by the small RNA *csrB*, which sequesters CsrA in response to environmental cues (Lee et al. 2019). Expression of *csrB* is controlled by the GrrS/GrrA (GacS/GacA or BarA/UvrY) two‐component system. Although no direct CsrA binding to *hrpL* or *hrpS* transcripts has been detected, CsrA regulates upstream factors such as *relA*, *rcsB* and *flhD* (Lee et al. 2019). Additional regulatory input is provided by the RcsBCD phosphorelay, which senses envelope stress and modulates *hrpS* expression. The Lon protease further ensures that regulatory proteins do not accumulate under non‐permissive conditions (Wang et al. 2009; Lee and Zhao 2018). The ClpXP protease indirectly influences T3SS by degrading the stationary‐phase sigma factor RpoS. In the absence of ClpXP, RpoS accumulates, resulting in a shift of bacterial transcription towards stress‐response by reducing T3SS expression and diminished virulence (Lee and Zhao 2017). The ribosome‐associated protein YhbH also contributes to efficient *hrpL* expression, linking translational capacity to RpoN‐dependent transcription (Ancona et al. 2014). At the level of the secretion apparatus, the proteins HrcU and HrpP are essential for proper T3SS function, and deletion of either results in markedly reduced virulence in immature pear assays (McNally et al. 2016). The second messenger c‐di‐GMP further modulates T3SS output. Specifically, elevated c‐di‐GMP levels repress *hrpA* (encoding a major pilus subunit) expression and it shifts bacterial behaviour towards biofilm formation while suppressing motility and T3SS activity (Edmunds et al. 2013; Kharadi et al. 2019, 2021b). Finally, the EnvZ/OmpR two‐component system negatively regulates *hrpL*, providing yet another layer of repression under specific environmental conditions (Li et al. 2014).

### 4.2 Exopolysaccharides: Amylovoran, Levan and Cellulose

EPSs play a vital role in the pathogenicity of *E. amylovora* by facilitating biofilm formation and obstructing the plant vascular system, thereby promoting colonisation and systemic spread (Koczan et al. 2011). The three major EPSs produced by *E. amylovora* , amylovoran, levan and cellulose, contribute differentially to virulence.

Amylovoran is the primary EPS, and mutants deficient in amylovoran production are nonpathogenic, underscoring its critical role (Koczan et al. 2009; Vrancken et al. 2013). Structurally, amylovoran is a heteropolymer composed of repeating units of glucose, galactose and pyruvate (Kharadi, Selbmann, and Sundin 2021; Nimtz et al. 1996). The biosynthesis of amylovoran is governed by the *ams* gene cluster, which includes 12 genes (*amsA*–*amsL*) that encode proteins with distinct functions in biosynthesis and export (Bugert and Geider 1995). Amylovoran biosynthesis is positively regulated by c‐di‐GMP through its modulation of RcsBCD phosphorelay and the c‐di‐GMP‐responsive ProQ/Prc pathway (Figure 2) (Yuan, Eldred, Kharadi, et al. 2022). In addition, its production is influenced by environmental factors such as pH, temperature and carbon sources (Edmunds et al. 2013). Conversely, the orphan regulator AmyR negatively regulates amylovoran production. Although the *amyR* mutant produces more amylovoran, its virulence does not exceed that of the wild type (Wang et al. 2012a). The additional details on amylovoran production are discussed under biofilm formation.

Levan, a neutral polyfructan (β‐2,6‐D‐fructofuranan), is synthesised from sucrose via the enzyme levansucrase, encoded by the *lsc* gene (Domżał‐Kędzia et al. 2023). While not essential for pathogenicity, levan contributes to biofilm structure and virulence. Its production is temperature‐dependent and positively regulated by the *rlsA* gene (Peng et al. 2021). The *lsc* gene is located downstream of *dspF* in the *dsp/hrp* region and is unrelated to the *ams* gene cluster (Zhang and Geider 1999).

Cellulose, composed of β‐1,4‐linked glucose units, is another key EPS that contributes to the three‐dimensional architecture of the biofilm (Castiblanco and Sundin 2018). Cellulose biosynthesis is regulated by c‐di‐GMP, which binds allosterically to the catalytic subunit BcsA (Figure 2). The enzyme BcsZ, an endoglucanase, is also essential for c‐di‐GMP‐mediated activation of cellulose biosynthesis (Domżał‐Kędzia et al. 2023).

### 4.3 Motility

Motility, mediated by peritrichous flagella, is a key virulence factor that facilitates both the initiation and progression of fire blight disease (Schachterle et al. 2019). It enables *E. amylovora* to actively migrate from the flower stigma to the hypanthium, a critical step for successful colonisation and infection. In the absence of motility, the pathogen fails to reach infection sites, resulting in a loss of virulence (Spinelli et al. 2005). Flagellar synthesis in *E. amylovora* is influenced by environmental conditions. Optimal flagellar production occurs at temperatures between 18°C and 25°C and pH range of 6–9 (Raymundo 1980). Although flagellar motility does not require an external energy source, it is enhanced in the presence of sugars such as mannitol and glucose. Notably, *E. amylovora* cells are typically non‐motile within host tissues but regain motility when exposed to free water and favourable environmental conditions (Raymundo 1980). Interestingly, once the pathogen has entered the apoplast, flagella appear to be dispensable. Studies have shown that flagella‐deficient mutants spread through young leaf tissues at rates comparable to wild‐type strains, suggesting that motility is primarily critical during the initial stages of infection and environmental navigation, rather than during intercellular colonisation (Holtappels et al. 2018). Recently, it has been shown that *E. amylovora* uses EPS‐dependent sliding motility to facilitate movement without flagella within the apoplast (Yuan, Eldred, and Sundin 2022), thus enabling the maintenance of a motile phenotype without presenting the flg22 epitope for plant recognition.

The regulation of motility in *E. amylovora* centres on the master regulator FlhDC, which controls transcription of the flagellar gene cascade and is frequently co‐expressed with T3SS‐associated genes (Puławska et al. 2017), highlighting the coordinated deployment of motility and virulence programmes (Figure S1). The RNA chaperone Hfq modulates *flhDC* expression through several RNAs, *arcZ*, *omrAB* and *rmaA*, which collectively fine‐tune swimming motility (Zeng and Sundin 2014). The *arcZ* and *omrAB* repress *flhDC* post‐transcriptionally, whereas *arcZ* and *rmaA* act as positive transcriptional regulators. The dual regulatory role of *arcZ*, particularly its positive influence on motility, appears to be unique to *E. amylovora* and may represent a recent evolutionary adaptation (Schachterle et al. 2019). The RcsCBD phosphorelay also integrates environmental signals to modulate *flhDC* expression. Within this system, the response regulator RcsB functions as a transcriptional repressor of *flhDC* (Lee et al. 2018). Post‐transcriptional control is further mediated by the global RNA‐binding protein CsrA, which enhances translation of both RcsB and FlhDC. CsrA activity is antagonised by the small RNA *csrB*, whose expression is positively regulated by the GrrS/GrrA two‐component system and by IHF (Ancona et al. 2016). Proteolytic regulation by Lon and ClpXP influences *flhDC* expression (Ancona et al. 2016; Lee and Zhao 2017). In Lon‐deficient strains, elevated RcsA/RcsB activity increases *hrpS* and *amsG* expression while reducing *flhD* and *csrB* levels; the resulting decrease in CsrB relieves sequestration of CsrA, indirectly enhancing CsrA‐dependent regulation (Ancona et al. 2016). Additional regulatory layers include the EnvZ/OmpR two‐component system, which positively influences flagellar motility, and the antagonistic activity of GrrS/GrrA towards EnvZ/OmpR (Li et al. 2014). The second messenger c‐di‐GMP acts as a potent negative regulator of swimming motility (Edmunds et al. 2013). Beyond repressing motility, c‐di‐GMP orchestrates the motility–biofilm transition: high intracellular levels promote biofilm formation, whereas low levels favour sliding motility, particularly during systemic movement through the xylem (Yuan, Eldred, and Sundin 2022). This balance is dynamically controlled by the activities of diguanylate cyclases and phosphodiesterases.

### 4.4 Biofilm Formation

Biofilm formation is a critical virulence strategy employed by *E. amylovora* to colonise host tissues and withstand environmental stress. Biofilms are structured multicellular communities embedded in a self‐produced extracellular matrix composed of EPS, proteins and nucleic acids (Hall and Mah 2017). This growth mode enhances bacterial survival, nutrient acquisition and resistance to antimicrobial agents (Danhorn and Fuqua 2007). Biofilm development follows a coordinated sequence of stages: planktonic phase, surface attachment, maturation and detachment (Sauer et al. 2002). Initial attachment is mediated by type I fimbrae and type IV pili, which anchor cells to host surfaces. Within the xylem vessels, *E. amylovora* forms dense biofilms that obstruct nutrient flow, contributing to vascular wilting, a hallmark of fire blight. All three major EPS, amylovoran, levan and cellulose, contribute to the biofilm matrix and structural integrity. Notably, the pathogen transitions between sessile and planktonic states throughout the infection cycle, especially during systemic spread (Koczan et al. 2011).

The bacterial second messenger c‐di‐GMP is a central regulator of biofilm formation in *E. amylovora* (shown in Figure 2) (Kharadi and Sundin 2019). Elevated intracellular c‐di‐GMP promotes EPS production and biofilm development, while simultaneously suppressing flagellar motility. Cellular c‐di‐GMP levels are determined by the activities of diguanylate cyclases (DGCs), which synthesise the molecule, and phosphodiesterases (PDEs) which degrade it (Jenal et al. 2017). Deletion of the DGCs, *edcC* and *edcE*, reduces c‐di‐GMP levels and impairs biofilm formation (Castiblanco and Sundin 2018). Conversely, deletion of two or more PDE genes (*pdeA*, *pdeB*, *pdeC*) increases intracellular c‐di‐GMP, enhancing amylovoran production and cell autoaggregation but reduces biofilm formation in liquid culture (Kharadi and Sundin 2019). A comprehensive 12‐gene deletion study further established c‐di‐GMP as a global regulator of lifestyle transitions and host colonisation in *E. amylovora* (Kharadi et al. 2022).

RNA chaperones also contribute to biofilm regulation through their influence on amylovoran and cellulose synthesis. Among Hfq‐dependent small‐RNAs, *arcZ* and *rprA* act as positive regulators of amylovoran production, whereas *omrAB* and *hrs6* act as negative regulators (Peng et al. 2021; Zeng et al. 2013). Although Hfq is known to promote biofilm formation in other plant pathogens such as *Xanthomonas campestris* and *Pantoea ananatis* , its precise role in *E. amylovora* remains to be fully elucidated (Jenal et al. 2017). The conserved RNA chaperone ProQ also modulates EPS biosynthesis, acting as a negative regulator of both amylovoran and cellulose (Figure 2). Deletion of *proQ* increases production of both polysaccharides and reduces *prc* mRNA levels, consistent with the *prc* promoter residing within the *proQ* locus. The periplasmic protease Prc and its partner lipoprotein NlpI negatively regulate amylovoran, whereas Spr (a Prc degraded peptidoglycan hydrolase) positively influences its synthesis. Genome‐wide transposon mutagenesis linked ProQ to c‐di‐GMP signalling, as *proQ* deletion elevated intracellular c‐di‐GMP and cellulose production, phenotypes reversed by deleting DGC‐encoding genes. In contrast, ProQ positively regulates transcripts encoding c‐di‐GMP PDEs through a mechanism independent of mRNA decay (Yuan, Eldred, Kharadi, et al. 2022).

Cyclic di‐GMP also indirectly influences transcription of *amsG*, the first gene of the *ams* operon, through binding to the EAL‐domain protein CsrD. CsrD and RNase E negatively regulate the small RNA (sRNA) *csrB*, which normally sequesters and inhibits the RNA‐binding protein CsrA (Kharadi and Sundin 2022). CsrA, in turn, positively regulates the RcsBCD phosphorelay. The Rcs system is a major activator of amylovoran biosynthesis, supported by the presence of an RcsB binding site in the *amsG* promoter (Wang et al. 2012b). RcsB acts as a direct transcriptional activator of the *ams* operon. In contrast, the Lon protease negatively regulates amylovoran production by modulating RcsA and RcsB levels; this repression is thought to be counteracted by CsrA (Lee et al. 2018). The ClpXP protease also promotes *amsG* expression by degrading the stationary‐phase sigma factor RpoS (Lee and Zhao 2017). Additionally, c‐di‐GMP positively regulates *amsG* expression through the peptidoglycan hydrolase EagA (Kharadi and Sundin 2020). Finally, the EnvZ/OmpR two‐component system, which also represses the T3SS, negatively regulates *amsG* and thereby suppresses amylovoran production and biofilm formation (Li et al. 2014).

### 4.5 Additional Virulence Determinants

Many other virulence determinants are encoded by *E. amylovora* and are important during various phases of the disease cycle. These include systems encoding the uptake and utilisation of major Rosaceae family transport sugars sorbitol and sucrose (Borruso et al. 2017; Piqué et al. 2015), the siderophore desferrioxamine that facilitates iron scavenging from the host environment, catalases KatA and KatG that counteract damage from oxidative stress arising mainly from host defences, and efflux pumps such as ArcAB and NorM that enable the bacterium to resist plant‐derived antimicrobial compounds and compete with other microbes in the host environment. Sulphur metabolism contributes to both oxidative stress management and energy production, which are vital for successful colonisation and disease progression. Transcriptomic profiling of *E. amylovora* during infection of apple flowers has revealed significant upregulation of several genes involved in sulphur assimilation and redox homeostasis, including *iscS*, *tpx*, *cysG*, *masA* and *dsbA*. These genes are associated with sulphur cycling and suggest an adaptive response to the oxidative environment encountered in host tissues (Schachterle et al. 2022).

In addition, the T6SS has remained relatively understudied but is emerging as an important contributor to the pathogen's virulence and ecological fitness. Genomic analysis has identified three distinct T6SS clusters in *E. amylovora* : T6SS‐1, T6SS‐2 and T6SS‐3 (Smits et al. 2010). The primary roles for T6SS in *E. amylovora* include bacterial competition, EPS production, virulence, motility and antibacterial activity (Tian et al. 2017). Transcriptomic analyses of T6SS deletion mutants have revealed differential expressions of key virulence‐associated genes, including those involved in T3SS, siderophore biosynthesis, chemotaxis, flagellar assembly and fimbrial structures (Kamber et al. 2017). This suggests that the T6SS may act as a regulatory hub, influencing multiple virulence pathways. Moreover, genomic islands associated with T6SS loci often contain genes encoding hallmark structural proteins such as HcP and VgrG, along with putative effector domains (De Maayer et al. 2011). These effectors may be involved in targeting competing microbes or modulating host exposure, although their specific functions in *E. amylovora* remain to be elucidated.

## 5 Regulatory Networks Governing Lifestyle Transitions in *E. amylovora*

The lifestyle transition of *E. amylovora* is tightly regulated by a complex network of signalling systems (summarised in Figure 2). Two‐component systems (TCSs) and c‐di‐GMP remain the center of this intricate regulatory system. TCSs in *E. amylovora* are an important sensor of environmental and host‐derived signals, enabling the pathogen to modulate virulence gene expression, motility and stress responses. Complementing these systems, c‐di‐GMP acts as a global regulator of lifestyle transitions. Elevated levels of c‐di‐GMP promote the switch from a motile planktonic state to a sessile biofilm‐forming phase, enhancing persistence and resistance to environmental stress (Edmunds et al. 2013) (summarised in Figure 2). Conversely, reduced c‐di‐GMP levels induce pathogen motility and T3SS expression, thereby favouring dispersal and active infection. The dynamic interplay between TCS‐mediated signal transduction and c‐di‐GMP metabolism allows *E. amylovora* to adapt rapidly to changing conditions within the host and the environment, balancing virulence and survival. Understanding these regulatory mechanisms is crucial for developing targeted strategies to disrupt biofilm formation and disease progression in agricultural settings.

Below, we summarise key TCSs and c‐di‐GMP signalling pathways in *E. amylovora* that contribute to its pathogenicity and lifestyle transitions. A detailed comparative analysis of TCSs and c‐di‐GMP among major species/strains from Enterobacterales order along with the evolutionary trajectories and functional diversification of these systems is explored in the subsequent section.

## 6 Major Two‐Component Systems in *E. amylovora*

Two‐component systems are central to the ability of *E. amylovora* to perceive and respond to environmental and host‐derived signals during infection. These systems typically consist of a membrane‐bound histidine kinase (HK) that detects specific stimuli and a corresponding response regulator (RR) that modulates gene expression. The genome of *E. amylovora* encodes 20 HK and 24 RR (Zhao et al. 2009) and a summary of selected major two‐component systems associated with virulence is summarised here to provide context for subsequent analyses of HK/RR evolutionary relationships and domain architecture.

Four major two‐component systems (RcsBCD phosphorelay—RcsC/RcsB, EnvZ/OmpR, GrrS/GrrA and HrpX/HrpY) contribute to virulence, motility and biofilm formation in *E. amylovora* . Among these, HrpX/HrpY functions exclusively in T3SS regulation, whereas RcsC/RcsB, EnvZ/OmpR and GrrS/GrrA integrate multiple signals to coordinate flagellar motility, T3SS activity and biofilm development. The HrpX/HrpY–HrpS–RpoN cascade is the core regulatory pathway controlling T3SS expression in *E. amylovora* and is conserved across other soft‐rot pectobacteria, including *Pectobacterium* and *Dickeya* spp. (Kvitko et al. 2025; Lee et al. 2016; Wei et al. 2007). In response to apoplast‐like cues such as low pH or nutrient limitation, HrpX phosphorylates HrpY, which activates the *σ* ^54^‐dependent enhancer‐binding protein HrpS. HrpS then stimulates RpoN (*σ* ^54^), initiating transcription of *hrpL*, the master regulator of T3SS genes (Wei et al. 2007). The RcsBCD phosphorelay (also referred to as RcsABCD, with RcsA acting as a non‐phosphorylating accessory protein) positively regulates both *hrpL* and *amsG*, thereby promoting T3SS expression and amylovoran‐mediated biofilm formation, while simultaneously repressing the flagellar master regulator *flhDC* (Wang et al. 2009, 2012b). The GrrS/GrrA system indirectly influences these pathways by activating the sRNA *csrB*, which sequesters CsrA and thereby enhances RcsBCD‐dependent regulation of biofilm formation, T3SS and motility (Ancona et al. 2016; Lee et al. 2019). EnvZ/OmpR exerts opposing effects on motility and virulence. OmpR activates *flhDC*, promoting flagellar motility (Li et al. 2014), but represses *hrpL* and *hrpN*, leading to reduced T3SS activity. OmpR also downregulates *amsG*, suppressing amylovoran production and biofilm formation (Li et al. 2014) (Figure 2). Together, these tw‐component systems form an integrated regulatory network that enables *E. amylovora* to fine‐tune motility, secretion and biofilm formation in response to dynamic host environments.

## 7 Cyclic di‐GMP in *E. amylovora* Associated With Virulence

Cyclic di‐GMP is a conserved bacterial second messenger that regulates the transition between motile and sessile lifestyles by coordinating motility, EPS production, surface adhesion and virulence traits (Figure 2) (Jenal et al. 2017; Römling et al. 2013). In *E. amylovora* , c‐di‐GMP promotes biofilm formation through upregulation of key biofilm matrix components amylovoran and cellulose, while concurrently repressing flagellar motility and T3SS gene expression. This regulatory shift facilitates transition from acute apoplast invasion to xylem associated colonisation (Edmunds et al. 2013). c‐di‐GMP is synthesised from two guanosine triphosphate (GTP) molecules by diguanylate cyclases (DGCs) containing GGDEF domains. Its degradation occurs via phosphodiesterase (PDEs) in a two‐step process: EAL or HD‐GYP domain containing PDE first linearise c‐di‐GMP into the intermediate molecule 5′‐phosphoguanylyl‐(3′,5′)‐guanosine (pGpG), which is subsequently hydrolysed into guanosine monophosphate (GMP) by oligoribonuclease or HD‐GYP PDEs. These enzymes are modulated by diverse effector proteins and allosteric regulators of EPS synthases (Anso et al. 2024). GGDEF domain proteins often possess additional sensory modules, enabling environmental cues to influence intracellular c‐di‐GMP levels (Hengge 2020). In *E. amylovora* , cellulose synthesis is activated by c‐di‐GMP through BcsA and supported by BcsZ, linking signal transduction to matrix polymer production (Castiblanco and Sundin 2018). EAL domain PDEs characterised by the conserved glutamate‐alanine‐leucine motif, frequently exhibit modular architectures with N‐terminal sensory domains that hydrolyse c‐di‐GMP into linear pGpG (Christen et al. 2007; Galperin 2001; Römling et al. 2013). HD‐GYP domain PDEs, though less common, belong to the HD superfamily of metal dependent phosphohydrolases and degrade c‐di‐GMP to GMP, contributing additional flexibility to the signalling network (Galperin et al. 1999; Stelitano et al. 2013). In *E. amylovora* , three EAL domain PDEs: PdeA, PdeC and PdeA/B/C, counterbalance DGC activity to fine‐tune biofilm formation. Deletion of these PDEs leads to increased surface attachment and EPS production, underscoring their role in regulating virulence and persistence (Römling et al. 2013; Ryan et al. 2009).

## 8 Evolutionary Perspective on Two‐Component Systems and c‐di‐GMP in *E. amylovora* With Respect to Other Enterobacterales

### 8.1 Phylogenetic and Domain Level Analysis of HKs and RRs

Two‐component systems in *E. amylovora* have likely evolved under selective pressures unique to its ecological niche and pathogenic lifestyle. Within the order Enterobacterales, species exhibit diverse virulence strategies and host adaptations, which are reflected in the evolutionary trajectories of their signal transduction systems (Capra and Laub 2012). To investigate this, we analysed the amino acid divergence of four key two‐component systems: RcsC/RcsB, EnvZ/OmpR, GrrS/GrrA and HrpX/HrpY across 13 bacterial species that are representative of four families (*Erwiniaceae*, *Pectobacteriaceae*, *Enterobacteriaceae* and *Yersiniaceae*) within Enterobacterales order, including two *E. amylovora* strains (Table S1).

**FIGURE 3:**
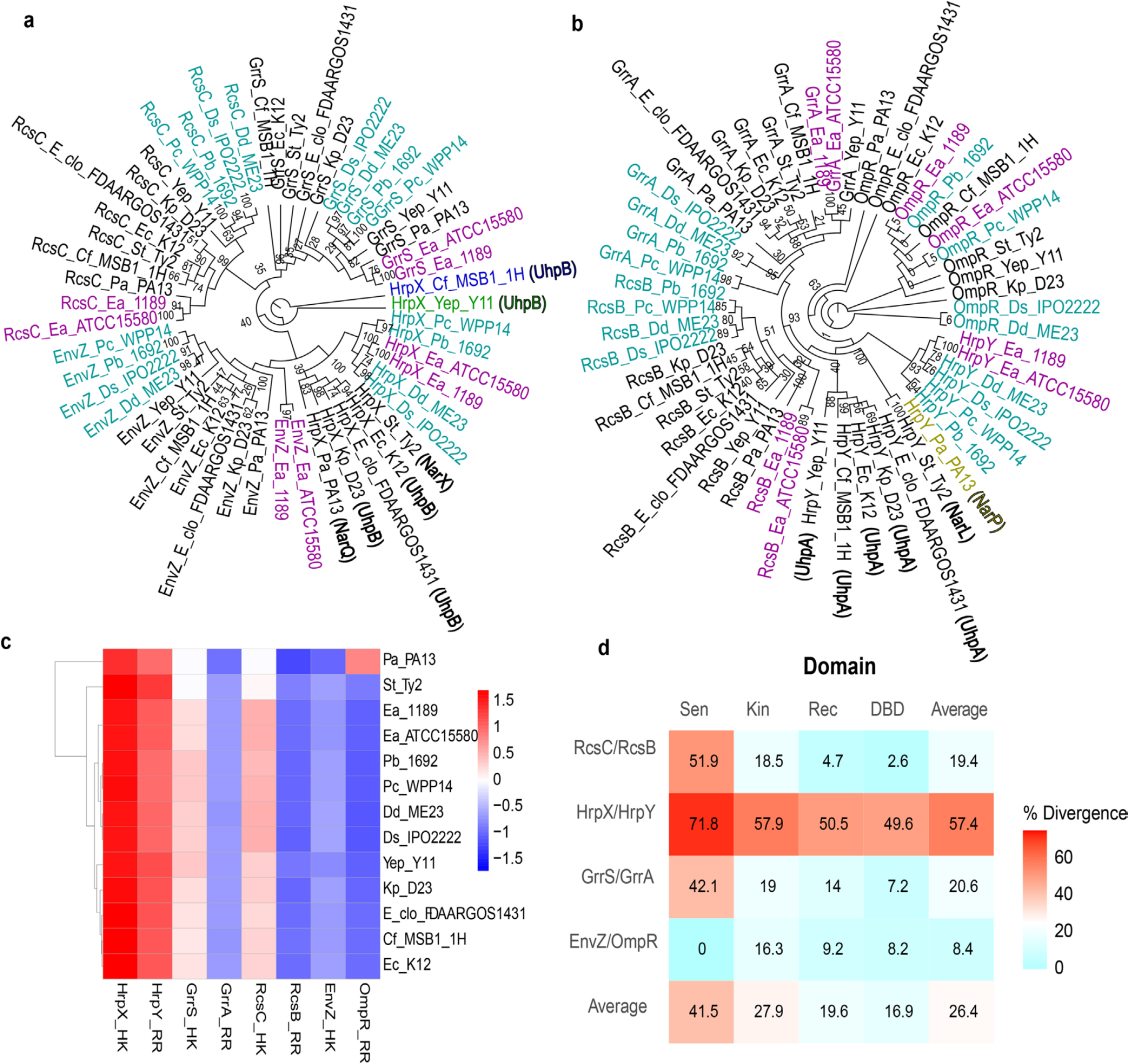
Phylogenetic relationships and domain level divergence analysis of HKs and RRs from 13 Enterobacterales species. (a) Maximum‐likelihood phylogenetic tree of HKs (RcsC, EnvZ, GrrS and HrpX). (b) Maximum‐likelihood phylogenetic tree of RRs (RcsB, OmpR, GrrA and HrpY). Each HK and RR formed distinct clades, reflecting their evolutionary separation. Pink, *Erwinia amylovora* strains 1189 and ATCC15580; cyan blue, *Pectobacterium* strains (Pb1692, WPP14) and *Dickeya* strains (ME23 and IPO2222); pale green, *Yersinia* strain Yep_Y11; brown, *Pantoea* strain PA13; blue‐, *Citrobacter* strain Cf MSB1_1H. The numbers at the nodes indicate bootstrap support value from 1000 iterations. Tree labels display gene IDs followed by the genus and species initials and the strain ID, with each element separated by an underscore. The protein IDs for HrpX and HrpY that have acquired different functional annotations are in the parentheses with bold letters. (c) Heatmap showing relative domain divergence across individual HKs (RcsC, EnvZ, GrrS and HrpX) and their cognate response regulators (RcsB, OmpR, GrrA and HrpY). The strain name is written using the genus and species initials followed by the strain ID. (d) Heatmap showing domain‐wise divergence patterns of HKs (sensor [Sen], kinase [Kin] and RRs, receiver [Rec], DNA‐binding domain [DBD]) across selected 13 bacterial strains. Sen and Kin are the HK domains and Rec and DBD are the RR domains. Divergence is expressed as amino acid substitutions per 100 residues.

*Erwinia amylovora* encodes 20 HKs and 24 RRs that collectively facilitate environmental sensing and regulation of virulence (Zhao et al. 2009). We performed phylogenetic analyses of selected HKs (RcsC, EnvZ, GrrS and HrpX) and RRs (RcsB, OmpR, GrrA and HrpY), previously identified as key virulence‐associated components (methods detailed in File S1). These analyses revealed the formation of distinct clades by each of the HKs and RRs (Figure 3a,b). Notably, HKs and RRs from *Pectobacterium* (Pb1692 and WPP14) and *Dickeya* (ME23 and IPO2222) (cyan blue colour text in Figure 3) grouped with HKs and RRs from *E. amylovora* (pink colour text in Figure 3), despite these organisms belonging to the *Pectobacteriaceae* family. Interestingly, the HrpX and cognate receptor HrpY from *Pectobacterium*, *Dickeya* and *Erwinia* species form distinct clades and diverged from other species from *Enterobacteriaceae* that formed a separate clade (Figure 3a,b). This phylogenetic proximity between HKs and RRs of *Pectobacterium*, *Dickeya* and *Erwinia* species suggests a shared ancestral origin, consistent with their historical classification under genus *Erwinia*. These genera may have retained conserved two‐component system prior to taxonomic divergence. Although it will be discussed in detail in the later section, it is worth mentioning here that HrpX and HrpY in other studied bacterial species have evolved to different functions that are written in the parentheses in phylogenetic trees in Figure 3a,b.

Among the two‐component systems analysed, HrpX/HrpY showed high divergence in both HK and RR domains (Figure S2a, Figure 3c). Domain level analysis revealed that sensor and kinase domains of HK were more divergent than the receiver and DNA‐binding domains of RR (Figure 3b), except for the sensor domain of EnvZ, which remained highly conserved probably due to its universal osmoregulation function (Aiba and Mizuno 1990). The high divergence of HrpX/HrpY reflects adaptation to host specific environmental cues such as pH, redox state and nutrient availability (Merighi et al. 2003). While HK domains evolve to accommodate diverse stimuli, RR domains remain conserved to maintain phosphorylation compatibility and DNA‐binding specificity (Capra and Laub 2012).

To quantify divergence, we compared the percentage amino acid divergence of HKs and RRs to 15 arbitrarily selected core genes (CG) across the 13 species (Table S1). HKs exhibited significantly higher divergence than CGs (Figure S2a), consistent with previous findings (Barretto et al. 2024). This elevated divergence likely reflects the need for HKs to recognise diverse external stimuli. RRs, in contrast, showed lower divergence rates, quite similar to CGs, suggesting conservation of intracellular signalling roles (Figure S2a) (Capra and Laub 2012). HKs often contain modular sensory domains (e.g., periplasmic domains, PAS, HAMP, GAF), which are highly variable and facilitate domain shuffling, enabling the evolution of novel sensing capabilities (Galperin et al. 2001). Heatmap analysis of pairwise divergence (Figure S2b,c) showed lower divergence values among *E. amylovora* , *Pectobacterium* and *Dickeya* strains, supporting their shared ancestry and similar necrotrophic lifestyles. These pathogens rely on similar functional two‐component systems such as HrpX/HrpY negatively regulates EPS production and biofilm formation, whereas positively regulates T3SS expression via activation of master regulator *hrpL* (Lee and Zhao 2018; Yap et al. 2008). The functional role of GrrS/GrrA, EnvZ/OmpR and RcsC/RcsB involved in virulence of these species are conserved. Overall, pairwise comparison of *E. amylovora* (*Ea*1189 and ATCC15580) to all human pathogens have divergence value greater than 100. Because this is a relative analysis to core genes, this observation supports the finding that human‐associated Enterobacterales split more recently (Baumler et al. 2013). These differences likely reflect distinct host interactions and virulence strategies. Notably, the pairwise divergence of HKs and RRs in *E. amylovora* (Ea1189 and ATCC15580) and plant pathogen *P. ananatis* PA13 was higher. *P. ananatis* PA13, although a plant pathogen within *Erwiniaceae*, employs toxin‐dependent necrotrophy, diverging from the T3Es and EPS‐based strategies of *E. amylovora* (Kvitko et al. 2025).

### 8.2 Modular Diversity in HKs Compared to Their RRs in *E. amylovora*

Histidine kinases in *E. amylovora* exhibited greater domain complexity and modularity than their cognate RR, reflecting their role in environmental signal detection and transduction. Typically, HKs possess between five and eight distinct domains whereas RRs are composed of only two: a receiver (Rec) domain and a DNA‐binding domain (DBD) (Figure S3). For example, RcsC and GrrS are hybrid HKs characterised by a sensor domain located between transmembrane helices, enabling detection of extracellular signals, followed by a conserved HisKA and HATPase domains responsible for autophosphorylation (Figure S3) (Rogov et al. 2006). These HKs also contain a C‐terminal Rec domain, indicative of a multi‐step phosphorelay mechanism. GrrS further exemplifies domain complexity by incorporating a HAMP domain at the N‐terminus, which facilitates signal transduction and a histidine phosphotransfer (HPT) domain at the C‐terminal, which mediates phosphate transfer to its cognate RR. EnvZ, a canonical HK, includes a sensor domain, HAMP, HK and HATPase domains functioning in osmoregulation through phosphorylation of OmpR (Yoshida et al. 2002). In contrast, HrpX represents one of the most divergent HKs identified in our analysis. In *E. amylovora* , as well as in *Pectobacterium* and *Dickeya* species, HrpX along with its cognate response regulator HrpY positively regulates the master regulator HrpL, which in turn activates T3SS genes. However, in other Enterobacterial members such as *Salmonella enterica* serovar Typhi Ty2, and *P. ananatis* PA13 HrpX/HrpY have evolved to NarX/NarL and NarQ/NarP TCSs respectively, both involved in responding to environmental nitrate and nitrite (Darwin and Stewart 1995). In Enterobacterales species such as *Enterobacter cochlea* FDAARGOS 1432, *Klebsiella pneumoniae* D23, *Escherichia coli* K121H, *Yersinia pestis* Y11 and *Citrobacter freundii* MsB1_1H, HrpX/HrpY has evolved to UhpB/UhpA TCS involved in uptake and utilisation of hexose phosphates (Weston and Kadner 1988). This evolutionary plasticity is further supported by studies showing that the HrpX/HrpY system has homologues in other bacterial taxa, such as VsrA/VsrD in *Ralstonia solanacearum* , DegS/DegU in *Bacillus subtilis* and UhpB/UhpA and NarX/NarP in *E. coli* (Wei et al. 2007). In contrast to HKs, RRs in *E. amylovora* display simpler architecture. Most contain a REC domain for phosphorylation and a DBD of the helix‐turn‐helix LuxR type (HTH‐LuxR), which is commonly associated with regulation of quorum sensing, virulence factors, motility and secretion (Brameyer and Heermann 2016). An exception is OmpR, whose DBD belongs to the Trans_reg_C family, characterised by a winged helix‐turn‐helix motif. The modularity and diversity of HKs underscore their evolutionary adaptability, allowing bacteria to integrate a wide range of environmental signals. In contrast, the conserved nature of RR domains reflects functional constraints required for maintaining phosphorylation compatibility and precise gene regulation.

### 8.3 Divergence and Specialisation of c‐di‐GMP Signalling Domains in *E. amylovora*

To understand the diversity and evolutionary adaptation of c‐di‐GMP, 12 well characterised c‐di‐GMP associated genes in *E. amylovora* were retrieved from NCBI, along with 30 c‐di‐GMP related genes, including 29 from *E. coli* K12 and one from *E. coli* 55189 (Kharadi et al. 2022; Povolotsky and Hengge 2016). Initially, BLASTp searches were performed using both sets of c‐di‐GMP protein sequences against protein sequences from 13 different strains within Enterobacterales in order to ensure comprehensive coverage and cross‐validation from all potential sequences. Hits containing c‐di‐GMP‐related domains were further analysed using the SMART domain identification tool (methods detailed in File S1). Through this process, we identified a collection of proteins across the 13 strains that contained the GGDEF domain, the EAL domain, or both domains (Table S2).

Within the Enterobacterales, *E. amylovora* has the lowest number of proteins harbouring c‐di‐GMP signalling domains, in contrast to human pathogens such as *E. coli* , *Enterobacter* and *Klebsiella* spp., which possess the highest diversity of these domains (Table S2). Similarly, members of the soft rot Pectobacteriaceae (SRP) also display reduced number of c‐di‐GMP signalling proteins. The expanded diversity observed in generalist and opportunistic species is hypothesised to support a more complex and flexible c‐di‐GMP regulatory network, potentially facilitating adaptation to a broader host range and environmental niches Schirmer (2016). In contrast, the limited number of c‐di‐GMP signalling proteins in host‐adapted pathogens such as *E. amylovora* , may reflect a streamlined signalling architecture tailored to its specialised host interactions. Despite the reduced number of c‐di‐GMP domain‐containing proteins, auxillary sensory domains, such as PAS, PAC, GAF, HAMP, BLUF, dCache, GAPES and MASE, were detected across all surveyed Enterobacterales species including *E. amylovora* . However, their abundance was positively correlated with the total number of c‐di‐GMP signalling proteins. The presence of these additional modules likely enhances signalling versatility, enabling integration of diverse environmental and intracellular cues into the c‐di‐GMP regulatory framework (Cruz et al. 2012; Hengge et al. 2016; Koppenhöfer and Lang 2022). Notably, most DGC and PDE domains exhibited high sequence diversity across strains, with many lacking definitive functional annotations, complicating predictive analyses. One exception was the degradation regulator protein CsrD (*yhdA*), which was conserved across all 13 strains examined. CsrD is catalytically inactive but plays a pivotal role in the turnover of small regulatory RNAs (CsrB and CsrC) via RNase E, thereby modulating the global carbon storage regulator system (CsrA‐CsrB/C) (Figure 2) (Romeo and Babitzke 2014). Although CsrD contains both GGDEF and EAL domains, it lacks the catalytic residues required for enzymatic activity. Instead, it functions as a regulatory adaptor linking c‐di‐GMP signalling to RNA degradation pathways. Specifically, the GG(D/E)EF motif in GGDEF domain is CsrD features a degenerate HRSDF motif (Chan et al. 2004), while EXLXR motif in EAL domain retains the conserved EXLXR motif essential for c‐di‐GMP binding (Barends et al. 2009; Minasov et al. 2009).

**FIGURE 4:**
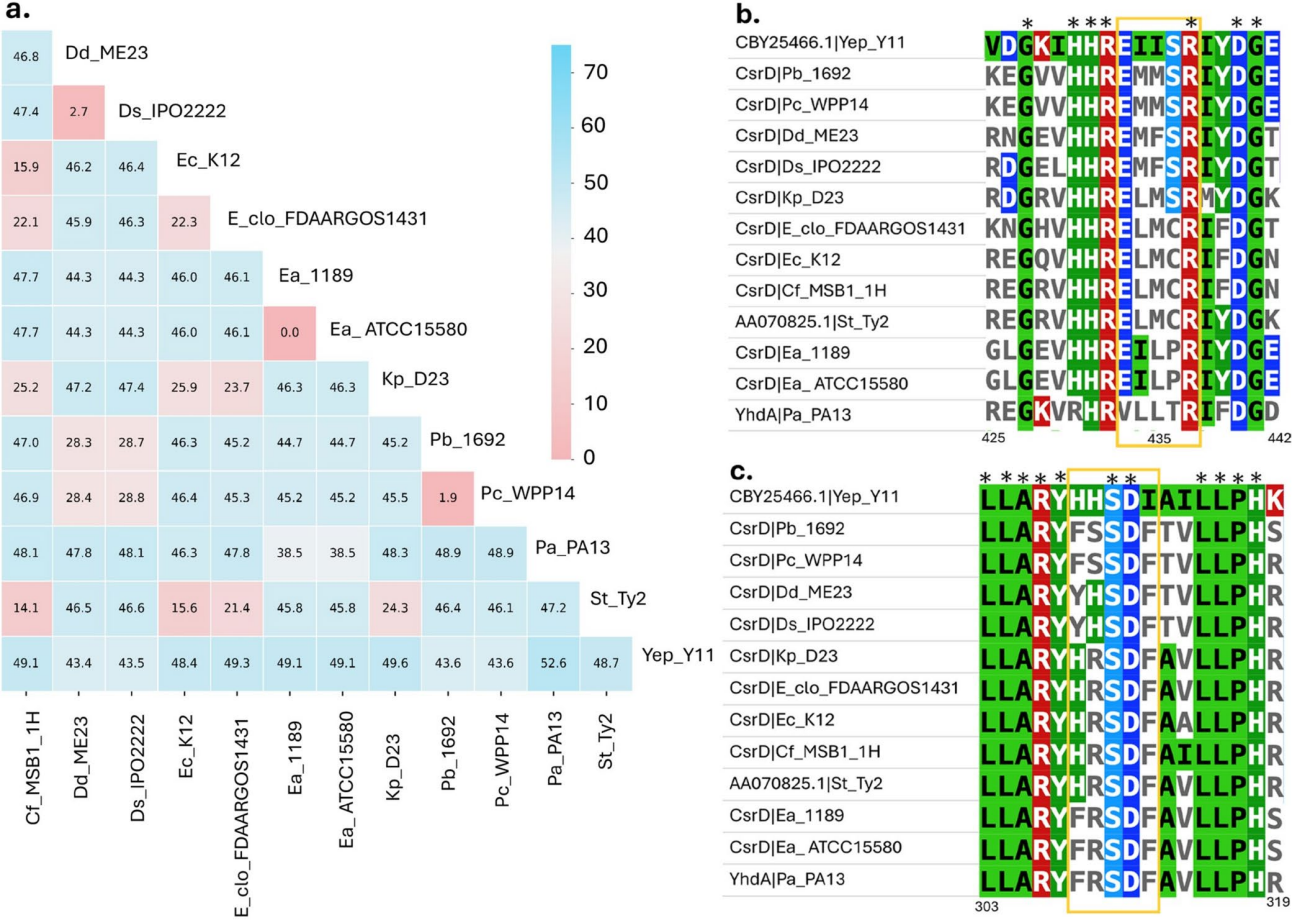
Variation in the CsrD protein across species within Enterobacterales order. (a) The heatmap represents pairwise amino acid divergence (%) of the CsrD protein among 13 Enterobacterales species. Each cell indicates the pairwise amino acid divergence between two strains, with lower values (pink) signifying lower amino acid divergence and higher values (blue) representing greater divergence. The two *Erwinia amylovora* strains (Ea_1189 and Ea_ATCC15580) have identical CsrD sequences (0% divergence), while showing low to moderate divergence (~35%–50%) across the genera. (b) Multiple sequence alignment of the EXLXR motif (highlighted) in EAL domain shows partial conservation, with arginine (R) residues retained, whereas the leucine (L) position is variable among species. (c) Alignment of the GGDEF domain shows a degenerate His‐Arg‐Ser‐Asp‐Phe (HRSDF) motif (highlighted) in CsrD orthologs. Only the serine (S) and aspartic acid (D) residues remained conserved across all strains.

In *E. amylovora* , the leucine residue at position 435 (L435) within the EAL domain of CsrD has been implicated in c‐di‐GMP binding, influencing RNase E‐mediated degradation of CsrB and promoting the production of amylovoran, biofilm formation and other virulence traits (Kharadi and Sundin 2022). To further investigate the evolutionary conservation and functional divergence of CsrD, multiple sequence alignment was performed across strains to assess pairwise amino acid divergence and identify conserved residues within active motif sites (Figure 4).

Amino acid divergence analysis revealed that CsrD is a relatively conserved protein across Enterobacterales, with pairwise divergence values predominantly ranging between 40% and 50%, suggesting functional conservation within this bacterial order (Figure 4a). Closely related species of *Pectobacterium* and *Dickeya* genera, as well as species or strains within the same genera, exhibited minimal divergence. In contrast, *Erwinia* strains were nearly identical, consistent with their close phylogenetic relationship. We further performed sequence alignment of the conserved motifs within EAL and GGDEF domains. EAL domain has EXLXR motif, where arginine residue (R437) conserved across all analysed Enterobacterales members (Figure 4b). The leucine residue (L435), previously known to mediate c‐di‐GMP binding and influence RNase E‐dependent turnover of CsrB (Kharadi and Sundin 2022), stayed the same in *Pantoea* and *Erwinia* species. This conservation likely reflects their close genetic relationship and shared pathogenic lifestyle, as c‐di‐GMP regulation plays a central role in biofilm formation and virulence (Bible et al. 2021). Divergence of L435 to M or F was observed in other Enterobacterales. Because these amino acids are nonpolar and hydrophobic, they might not have a strong chemical consequence to the EXLXR motif.

The GGDEF domain has HRSDF motif, of which serine (S) and aspartic acid (D) residues are conserved across all tested Enterobacterales (Figure 4c). Other residues are poorly conserved across Enterobacterales, similar to observations made in *Salmonella* ATM3375 where the homologue of CsrD has been referred to as having additional activity of binding c‐di‐GMP or other related nucleotide substrates (Jonas et al. 2010). The domain might have acquired RNA binding functionality from the Palm domain family of nucleotidyltransferases and RNA‐binding proteins (Pei and Grishin 2001; Römling et al. 2013), consistent with its role in RNase E mediated degradation of CsrB and CsrC. Given the structural similarity between c‐di‐GMP and small circular RNAs (Suzuki et al. 2006), this represents an evolutionary trajectory from RNA binding origin to diguanylate cyclase activity, and subsequently to RNA regulatory function. This divergence of CsrD from canonical diguanylate cyclases and phosphodiesterases, and its evolution into a non‐enzymatic regulatory adaptor, underscores the remarkable plasticity of the c‐di‐GMP signalling network across Enterobacterales. This functional repurposing highlights the adaptive potential of signalling proteins in response to ecological and pathogenic pressures.

## 9 Conclusion and Future Prospects

*Erwinia amylovora* employs a sophisticated array of virulence factors to initiate and sustain infection, beginning with the early priming of the T3SS during its epiphytic phase, and progressing to endophytic invasion and systemic colonisation of the apoplast and vascular tissues. A critical aspect of the pathogenic strategy of *E. amylovora* is the transition from a motile, planktonic lifestyle to a sessile, biofilm‐associated state. This transition is governed by a complex regulatory network. Among the regulatory networks, four two‐component systems discussed in this review play vital roles in both virulence and lifestyle transitions. While several two‐component systems such as GrrS/GrrA, RcsC/RcsB and EnvZ/OmpR exhibit functional conservation across Enterobacterales species, HrpX/HrpY stands out due to its functional and sequence divergence in related genera such as *Pantoea*, *Citrobacter* and other human‐associated pathogens.

Despite being identified over a century ago, many aspects of *E. amylovora* biology remain unresolved. The contribution of non‐host and alternate host plants to pathogen persistence and dissemination is still poorly defined. Several T3SS‐associated proteins, including effectors such as Eop2 and secreted components like HrpK, remain functionally uncharacterised. Although the mechanisms underlying biofilm formation are increasingly well understood, the regulatory cues and molecular machinery governing biofilm dispersal are largely unknown. Similarly, tT6SS, now recognised as an important determinant of virulence and ecological competitiveness, has only recently begun to receive attention in this species.

A major gap in our understanding lies in the evolutionary dynamics of virulence genes and their regulatory networks. Comparative analyses across Enterobacterales suggest that lifestyle transitions, host specialisation, and ecological pressures have shaped the diversification of key regulatory systems, yet these trajectories remain underexplored for *E. amylovora* . Future research integrating functional genomics, evolutionary biology and ecological studies will be essential for unravelling how regulatory circuits such as c‐di‐GMP signalling and two‐component system pathways have evolved and how they contribute to host adaptation, environmental persistence and disease emergence. Advancing this knowledge will not only deepen our understanding of plant pathogenicity but also support the development of more targeted and sustainable strategies for managing fire blight across diverse agricultural landscapes.

## Author Contributions

**Dhirendra Niroula:** conceptualization, methodology, formal analysis, visualisation, writing – original draft. **Kalyani Bhandari:** conceptualization, methodology, formal analysis, visualisation, writing – original draft. **George W. Sundin:** conceptualization, Validation, Supervision, writing – review and editing. **Janak R. Joshi:** conceptualization, validation, supervision, funding acquisition, writing – review and editing.

## Conflicts of Interest

The authors declare no conflicts of interest.

## Supporting information


**Figure S1:** Regulatory network controlling flagellar motility in *Erwinia amylovora* . This figure illustrates the multilayered regulatory circuitry that controls flagellar motility in *E. amylovora* . The master regulator FlhDC activates transcription of flagellar genes and is tightly coordinated with virulence‐associated pathways. FlhDC expression is influenced by global regulators, two‐component systems, small RNAs, proteolytic control and c‐di‐GMP signalling. Post‐transcriptional regulation is mediated by the RNA chaperone Hfq together with the sRNAs ArcZ, OmrAB and RmaA. ArcZ and OmrAB repress *flhDC*, while ArcZ and RmaA also enhance *flhDC* transcription. The Rcs phosphorelay (RcsABCD), particularly RcsB, functions as a negative regulator of *flhDC*. CsrA promotes expression of both *flhDC* and *rcsB*, but its activity is counteracted by the sRNA CsrB, whose transcription is stimulated by the GrrS/GrrA two‐component system and integration host factor. The Lon protease modulates levels of FlhDC and RcsBCD, indirectly shaping CsrA activity. EnvZ/OmpR positively regulates *flhDC*, whereas GrrS/GrrA antagonises EnvZ/OmpR signalling. Green arrows denote positive regulatory interactions, red bars indicate negative regulation and dashed lines represent Hfq‐dependent sRNA interactions.

**Figure S2:** Comparative divergence of HKs and RRs relative to core genes (CG) across Enterobacterales. (a) The average percentage amino acid divergence (amino acid substitution per 100 amino acids) across all proteins analysed and all pairwise combinations of reference strains for histidine kinases (HKs), response regulators (RRs) and the conserved gene (CG) set. Error bars indicate the standard deviation. Statistical significance of divergence rates of HKs and RRs compared to CGs was assessed by one‐way ANOVA; ** indicates *p* < 0.001, n.s.s. indicates not statistically significant. (b, c) Heat maps showing divergence rates of HKs (b) and RRs (c) relative to expected values across all strain pairs. Expected divergence for a given pair was calculated by scaling the average CG divergence for that pair using a correction factor to account for differences in divergence rates between CGs and HKs (b) or RRs (c). Values are expressed as percentages, with > 100% indicating greater‐than‐expected divergence and < 100% indicating lower‐than‐expected divergence. For pairwise comparisons, the strain name is written using the genus and species initials followed by the strain designation.

**Figure S3:** Domain architecture of histidine kinases (HKs) and response regulators (RRs) in four representative two‐component systems (TCSs) in *Erwinia amylovora* . Schematic representation of domain organisation in the HKs (RcsC, EnvZ, GrrS and HrpX) and their cognate RRs (RcsB, OmpR, GrrA and HrpY). Conserved domains are indicated by distinct shapes: PAS/PAC (signal sensing), HAMP (linker), HisKA (histidine kinase), HATPase_c (ATP‐binding), REC (receiver), HPT (histidine phosphotransferase) and DNA‐binding domains (HTH_LuxR or Trans_reg_C). The diversity of sensory and regulatory modules reflects the variation in environmental sensing and signal transduction across the studied TCSs.

**File S1:** mpp70228‐sup‐0004‐FileS1.docx.

**Table S1:** Representative bacteria from Enterobacterales order used in in silico comparative proteome analysis.

**Table S2:** Comparative distribution of c‐di‐GMP turnover domains (GGDEF, and EAL) across species within the Enterobacterales order.

## Data Availability

All protein sequences used in this study have been deposited in a Figshare repository https://figshare.com/articles/dataset/Virulence_Regulation_and_Lifestyle_Transitions_The_role_of_two‐component_systems_in_i_Erwinia_amylovora_i_and_its_evolutionary_context_within_Enterobacterales/30359002.
