# Supplementary material for: Virulence Regulation and Lifestyle Transitions: The Role of c‐di‐GMP and Two‐Component Systems in Erwinia amylovora and Their Evolutionary Context Within Enterobacterales
Source: Mol Plant Pathol. 2026 Feb 16;27(2):e70228. doi: 10.1111/mpp.70228 (PMC12910134; doi:10.1111/mpp.70228)
Supplement: Supplementary file 1 — Figure S1: Regulatory network controlling flagellar motility in Erwinia amylovora . This figure illustrates the multilayered regulatory circuitry that controls flagellar motility in E. amylovora . The master regulator FlhDC activates transcription of flagellar genes and is tightly coordinated with virulence‐associated pathways. FlhDC expression is influenced by global regulators, two‐component systems, small RNAs, proteolytic control and c‐di‐GMP signalling. Post‐transcriptional regulation is mediated by the RNA chaperone Hfq together with the sRNAs ArcZ, OmrAB and RmaA. ArcZ and OmrAB repress flhDC, while ArcZ and RmaA also enhance flhDC transcription. The Rcs phosphorelay (RcsABCD), particularly RcsB, functions as a negative regulator of flhDC. CsrA promotes expression of both flhDC and rcsB, but its activity is counteracted by the sRNA CsrB, whose transcription is stimulated by the GrrS/GrrA two‐component system and integration host factor. The Lon protease modulates levels of FlhDC and RcsBCD, indirectly shaping CsrA activity. EnvZ/OmpR positively regulates flhDC, whereas GrrS/GrrA antagonises EnvZ/OmpR signalling. Green arrows denote positive regulatory interactions, red bars indicate negative regulation and dashed lines represent Hfq‐dependent sRNA interactions. [file MPP-27-e70228-s006.docx]

**
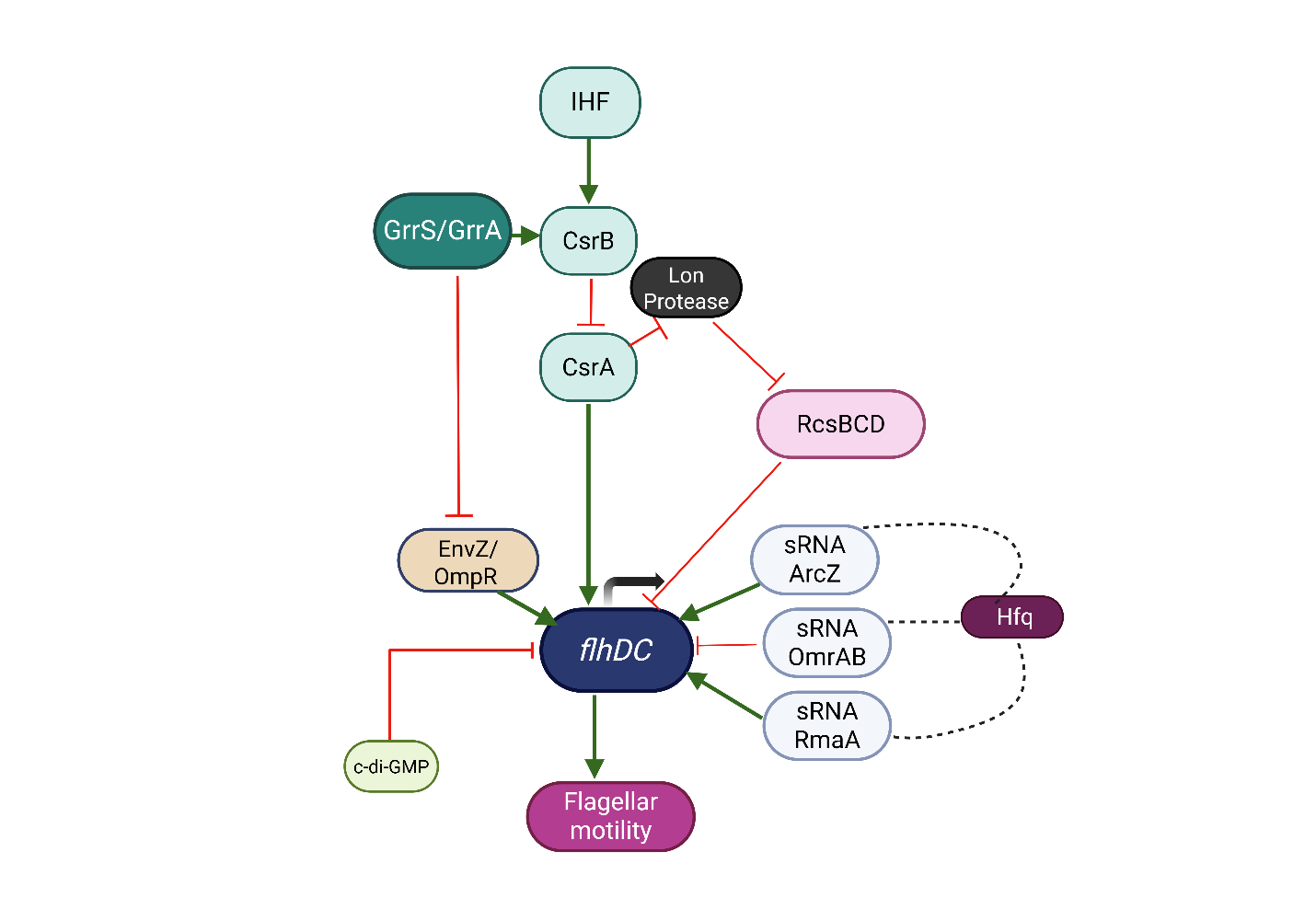
**

**Supplementary Figure S1: Regulatory network controlling flagellar motility in *E. amylovora*.** This figure illustrates the multilayered regulatory circuitry that controls flagellar motility in *E. amylovora*. The master regulator FlhDC activates transcription of flagellar genes and is tightly coordinated with virulence‑associated pathways. FlhDC expression is influenced by global regulators, two‑component systems, small RNAs, proteolytic control, and c‑di‑GMP signaling. Post‑transcriptional regulation is mediated by the RNA chaperone Hfq together with the sRNAs ArcZ, OmrAB, and RmaA. ArcZ and OmrAB repress *flhDC*, while ArcZ and RmaA also enhance *flhDC* transcription. The Rcs phosphorelay (RcsABCD), particularly RcsB, functions as a negative regulator of *flhDC*. CsrA promotes expression of both *flhDC* and *rcsB*, but its activity is counteracted by the sRNA CsrB, whose transcription is stimulated by the GrrS/GrrA two‑component system and integration host factor. The Lon protease modulates levels of FlhDC and RcsBCD, indirectly shaping CsrA activity. EnvZ/OmpR positively regulates *flhDC*, whereas GrrS/GrrA antagonizes EnvZ/OmpR signaling. Green arrows denote positive regulatory interactions, red bars indicate negative regulation, and dashed lines represent Hfq‑dependent sRNA interactions.
