# Supplementary material for: Virulence Regulation and Lifestyle Transitions: The Role of c‐di‐GMP and Two‐Component Systems in Erwinia amylovora and Their Evolutionary Context Within Enterobacterales
Source: Mol Plant Pathol. 2026 Feb 16;27(2):e70228. doi: 10.1111/mpp.70228 (PMC12910134; doi:10.1111/mpp.70228)
Supplement: Supplementary file 2 — Figure S2: Comparative divergence of HKs and RRs relative to core genes (CG) across Enterobacterales. (a) The average percentage amino acid divergence (amino acid substitution per 100 amino acids) across all proteins analysed and all pairwise combinations of reference strains for histidine kinases (HKs), response regulators (RRs) and the conserved gene (CG) set. Error bars indicate the standard deviation. Statistical significance of divergence rates of HKs and RRs compared to CGs was assessed by one‐way ANOVA; ** indicates p < 0.001, n.s.s. indicates not statistically significant. (b, c) Heat maps showing divergence rates of HKs (b) and RRs (c) relative to expected values across all strain pairs. Expected divergence for a given pair was calculated by scaling the average CG divergence for that pair using a correction factor to account for differences in divergence rates between CGs and HKs (b) or RRs (c). Values are expressed as percentages, with > 100% indicating greater‐than‐expected divergence and < 100% indicating lower‐than‐expected divergence. For pairwise comparisons, the strain name is written using the genus and species initials followed by the strain designation. [file MPP-27-e70228-s004.docx]

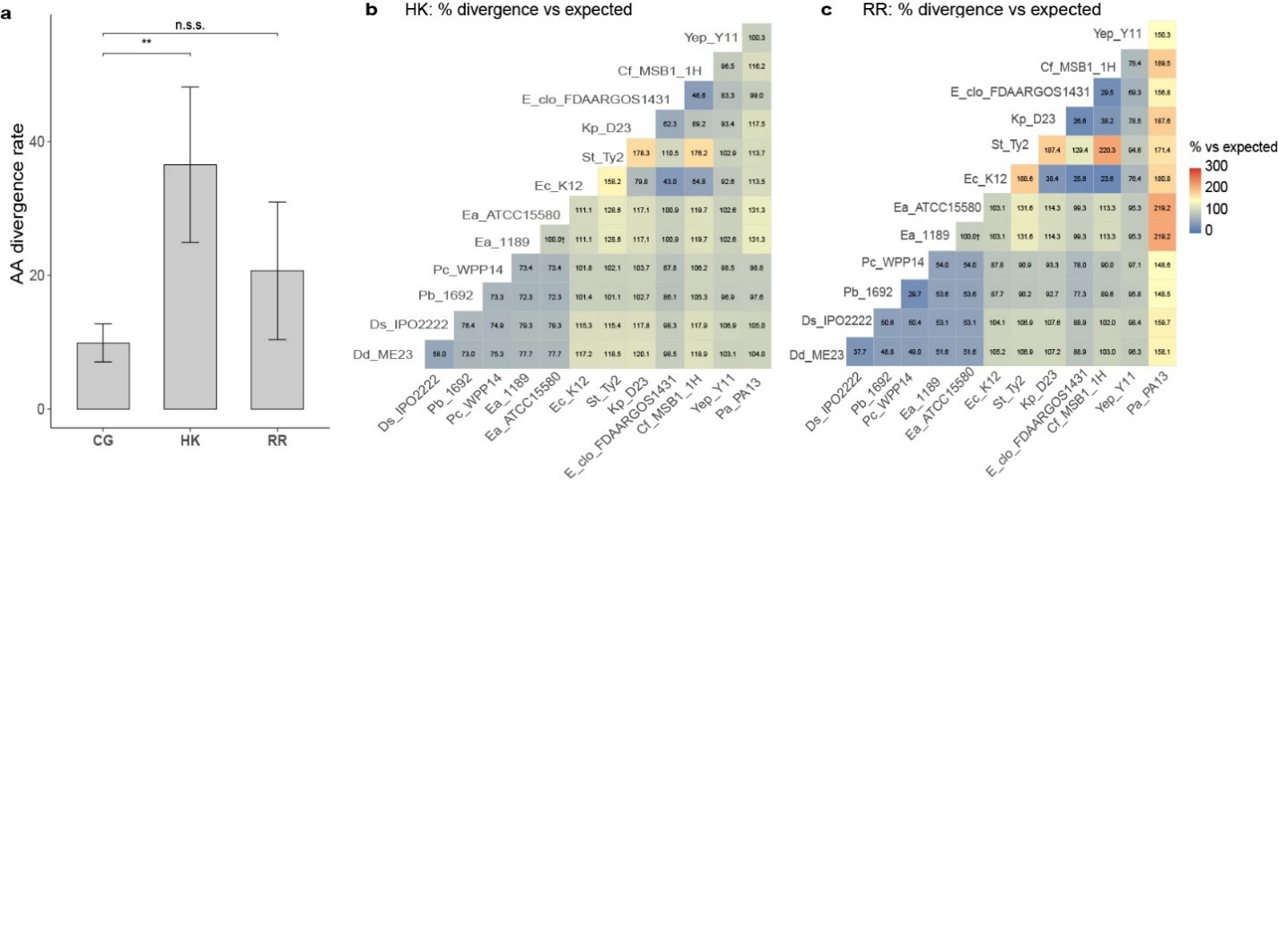


**Supplementary Figure S2: Comparative divergence of HKs and RRs relative to core genes (CG) across Enterobacterales**. (a) The average percentage amino acid divergence (amino acid substitution per 100 amino acids) across all proteins analyzed and all pairwise combinations of reference strains for histidine kinases (HKs), response regulators (RRs), and the conserved gene (CG) set. Error bars indicate the standard deviation. Statistical significance of divergence rates of HKs and RRs compared to CGs was assessed by one-way ANOVA; ** indicates P < 0.001, n.s.s. indicates not statistically significant. (b–c) Heat maps showing divergence rates of HKs (b) and RRs (c) relative to expected values across all strain pairs. Expected divergence for a given pair was calculated by scaling the average CG divergence for that pair using a correction factor to account for differences in divergence rates between CGs and HKs (b) or RRs (c). Values are expressed as percentages, with >100% indicating greater-than-expected divergence and <100% indicating lower-than-expected divergence. For pairwise comparisons, the strain name is written using the genus and species initials followed by the strain designation.
