# Supplementary material for: Virulence Regulation and Lifestyle Transitions: The Role of c‐di‐GMP and Two‐Component Systems in Erwinia amylovora and Their Evolutionary Context Within Enterobacterales
Source: Mol Plant Pathol. 2026 Feb 16;27(2):e70228. doi: 10.1111/mpp.70228 (PMC12910134; doi:10.1111/mpp.70228)
Supplement: Supplementary file 3 — Figure S3: Domain architecture of histidine kinases (HKs) and response regulators (RRs) in four representative two‐component systems (TCSs) in Erwinia amylovora . Schematic representation of domain organisation in the HKs (RcsC, EnvZ, GrrS and HrpX) and their cognate RRs (RcsB, OmpR, GrrA and HrpY). Conserved domains are indicated by distinct shapes: PAS/PAC (signal sensing), HAMP (linker), HisKA (histidine kinase), HATPase_c (ATP‐binding), REC (receiver), HPT (histidine phosphotransferase) and DNA‐binding domains (HTH_LuxR or Trans_reg_C). The diversity of sensory and regulatory modules reflects the variation in environmental sensing and signal transduction across the studied TCSs. [file MPP-27-e70228-s005.docx]

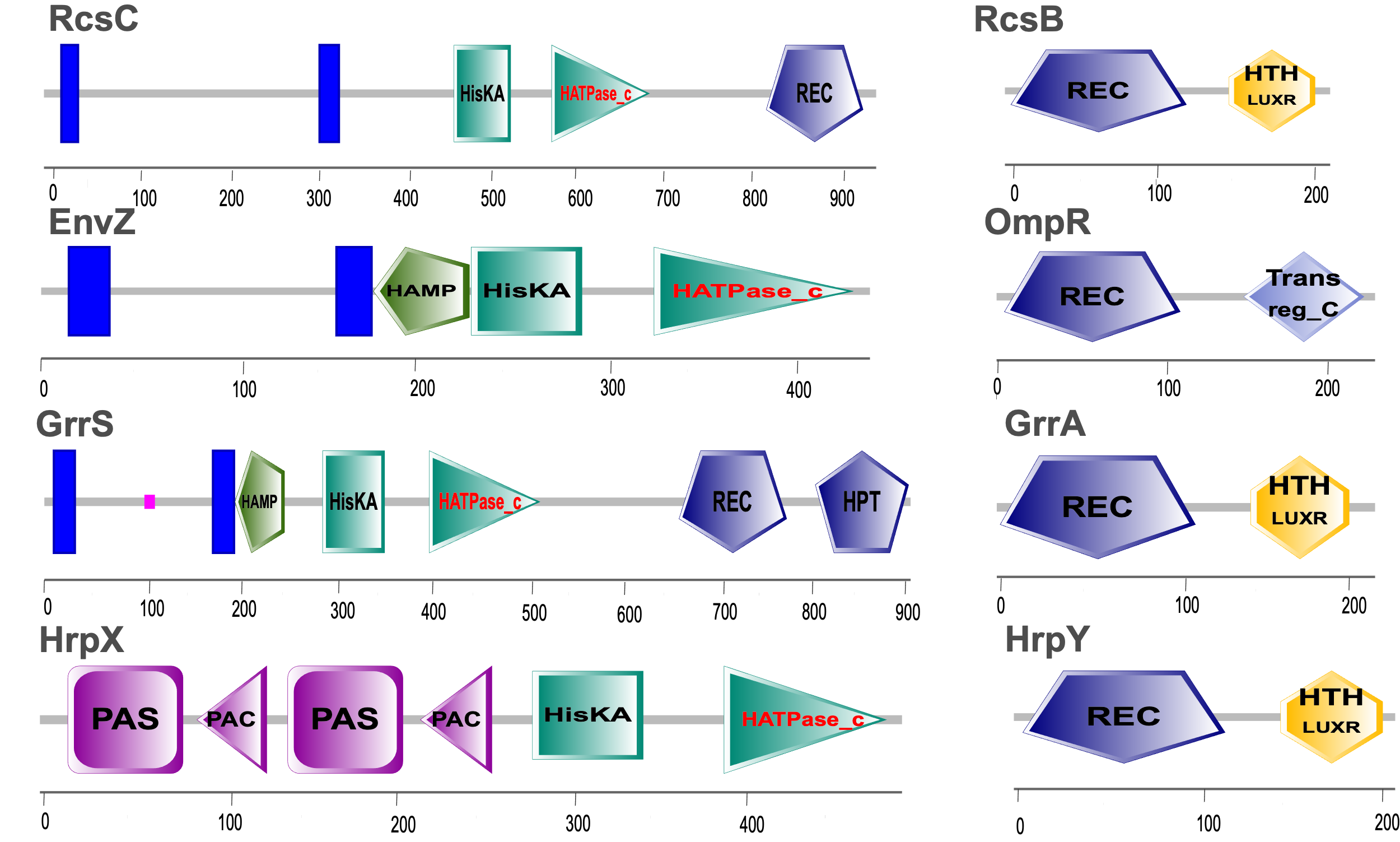


**Supplementary Figure S3: Domain architecture of histidine kinases (HKs) and response regulators (RRs) in four representative two-component systems (TCSs) in *Erwinia amylovora*.** Schematic representation of domain organization in the HKs (RcsC, EnvZ, GrrS, and HrpX) and their cognate RRs (RcsB, OmpR, GrrA, and HrpY). Conserved domains are indicated by distinct shapes: PAS/PAC (signal sensing), HAMP (linker), HisKA (histidine kinase), HATPase_c (ATP-binding), REC (receiver), HPT (histidine phosphotransferase), and DNA-binding domains (HTH_LuxR or Trans_reg_C). The diversity of sensory and regulatory modules reflects the variation in environmental sensing and signal transduction across the studied TCSs.
