# Supplementary material for: Virulence Regulation and Lifestyle Transitions: The Role of c‐di‐GMP and Two‐Component Systems in Erwinia amylovora and Their Evolutionary Context Within Enterobacterales
Source: Mol Plant Pathol. 2026 Feb 16;27(2):e70228. doi: 10.1111/mpp.70228 (PMC12910134; doi:10.1111/mpp.70228)
Supplement: Supplementary file 4 — File S1: mpp70228‐sup‐0004‐FileS1.docx. [file MPP-27-e70228-s001.docx]

**Supplementary methods:**

**Methods for Two Component System (TCS) Sequence Analysis:**

The Proteome sequences from 13 Enterobacterales species were retrieved from NCBI (Katoh and Standley 2013). TCS proteins from *Erwinia*, *Pectobacterium*, and *Dickeya* species were selected to build a custom database, which was then queried using BLAST (Mahram and Herbordt 2015) against the full proteomes of the selected strains (Supplementary Table S1). For phylogenetic analysis, Histidine Kinase (HK) and Response Regulator (RR) sequences were aligned using MAFFT v7.520 (Katoh and Standley 2013). Phylogenetic trees were constructed using RAxML with the LG+G8+F substitution model and 1000 bootstrap replicates (Kozlov et al. 2019) and visualized in FigTree v1.4.4 (Rambaut 2010).

Global alignments of CG, HK, and RR sequences were performed using the Biostrings R package with the BLOSUM62 substitution matrix (Bodenhofer et al. 2015). Percentage identity matrices were generated, and divergence values were calculated. Divergence values for HKs and RRs were normalized to CG baselines to compute expected divergence, and heatmaps of pairwise divergence across the 13 species were generated using ggplot2. Relative amino acid divergence was calculated as the mean divergence per strain (average divergence of each strain compared to all others). For domain-level divergence analysis, Sen, Kin, Rec, and DBD domains were identified using SMART, InterPro, and NCBI CD-Search (Marchler-Bauer et al. 2015). Divergence matrices and corresponding heatmaps were generated using the aforementioned tools.

**Methods for cyclic-di-GMP sequence analysis:**

To investigate c-di-GMP signaling proteins, BLASTp searches (Mahram and Herbordt 2015) were performed using reference sequences from *Erwinia amylovora* and *Escherichia coli* against proteomes of 13 representative Enterobacterales strains. Candidate proteins containing c-di-GMP-related domains were validated using the SMART database (Schultz 2000) to confirm domain architecture, and proteins containing GGDEF, EAL, or both domains were cataloged. For detailed analysis of the CsrD domain, multiple sequence alignments were conducted using MAFFT v7.520 (Katoh and Standley 2013), followed by pairwise amino acid divergence calculations. Conserved motif regions and residues across the 12 strains were visualized and aligned using Clustal Omega (Sievers and Higgins 2014), enabling identification of conserved and variable regions within the domain.

**References:**

Bodenhofer, U., Bonatesta, E., Horejš-Kainrath, C., and Hochreiter, S. 2015. msa: an R package for multiple sequence alignment. Bioinformatics 31:3997–3999.

Katoh, K., and Standley, D. M. 2013. MAFFT Multiple Sequence Alignment Software Version 7: Improvements in performance and usability. Molecular Biology and Evolution 30:772–780.

Kozlov, A. M., Darriba, D., Flouri, T., Morel, B., and Stamatakis, A. 2019. RAxML-NG: a fast, scalable and user-friendly tool for maximum likelihood phylogenetic inference. Bioinformatics 35:4453–4455.

Mahram, A., and Herbordt, M. C. 2015. NCBI BLASTP on High-performance reconfigurable computing systems. ACM Translocations on Reconfigurable Technology and Systems 7:1–20.

Marchler-Bauer, A., Derbyshire, M. K., Gonzales, N. R., Lu, S., Chitsaz, F., Geer, L. Y., Geer, R. C., He, J., Gwadz, M., Hurwitz, D. I., Lanczycki, C. J., Lu, F., Marchler, G. H., Song, J. S., Thanki, N., Wang, Z., Yamashita, R. A., Zhang, D., Zheng, C., and Bryant, S. H. 2015. CDD: NCBI’s conserved domain database. Nucleic Acids Research 43: D222–D226.

Rambaut, A. 2010. FigTree v1. 3.1 (http://tree. bio. ed. ac. uk/software/figtree/) Institute of Evolutionary Biology. University of Edinburgh, Edinburgh, United Kingdom.

Schultz, J. 2000. SMART: a web-based tool for the study of genetically mobile domains. Nucleic Acids Research 28:231–234.

Sievers, F., and Higgins, D. G. 2014. Clustal Omega. Current Protocols in Bioinformatics 48.
