# Supplementary material for: Virulence Regulation and Lifestyle Transitions: The Role of c‐di‐GMP and Two‐Component Systems in Erwinia amylovora and Their Evolutionary Context Within Enterobacterales
Source: Mol Plant Pathol. 2026 Feb 16;27(2):e70228. doi: 10.1111/mpp.70228 (PMC12910134; doi:10.1111/mpp.70228)
Supplement: Supplementary file 5 — Table S1: Representative bacteria from Enterobacterales order used in in silico comparative proteome analysis. [file MPP-27-e70228-s003.docx]

**Supplementary Table S1:** Representative bacteria from Enterobacterales order used in insilico comparative proteome analysis.

| **Bacterial strains** | **Acronyms** | **Family** | **Disease caused** |
| --- | --- | --- | --- |
| *Erwinia amylovora* strain Ea1189 | Ea_1189 | Erwiniaceae | Cause Fire blight in plants in Roseaceae including Apples and Pears |
| *Erwinia amylovora* strain ATCC15580 | Ea_ATCC15580 | Erwiniaceae | Cause Fire blight in plants in Roseaceae including Apples and Pears |
| *Pantoea annatis* strain PA13 | Pa_PA13 | Erwiniaceae | Cause Rice sheath rot and grain rot in rice. Also causes leaf spot in corn and onions |
| *Pectobacterium carotovorum* strain WPP14 | Pc_WPP14 | Pectobacteriaceae | Cause black leg and soft rot disease in Solanaceous plants including potato |
| *Pectobacterium brasiliense* strain Pb1692 | Pb_1692 | Pectobacteriaceae | Cause black leg and soft rot disease in Solanaceous plants including potato |
| *Dickeya dianthicola* strain ME23 | Dd_ME23 | Pectobacteriaceae | Cause black leg and soft rot disease in Solanaceous plants including potato |
| *Dickeya solani* strain IPO2222 | Ds_IPO2222 | Pectobacteriaceae | Cause black leg and soft rot disease in Solanaceous plants including potato |
| *Escherichia coli* strain K12 | Ec_K12 | Enterobacteriaceae | Nonpathogenic laboratory model strain |
| *Enterobacter cochlae* strain FDAARGOS 1431 | E_clo_FDAARGOS1431 | Enterobacteriaceae | Opportunistic pathogen causes nosocomial infection in human |
| *Salmonella enterica* serovar Typhi strain Ty2 | St_Ty2 | Enterobacteriaceae | Cause typhoid fever in human |
| *Citrobacter ferundii* MSB1 1H | Cf_MSB1_1H | Enterobacteriaceae | Cause a range of nosocomial and opportunistic infections such as urinary tract infections, respiratory infections, bacteremia, meningitis, and wound infections |
| *Klebsiella pneumoniae* strain D23 | Kp_D23 | Enterobacteriaceae | Cause nosocomial infection including, pneumonia, urinary tract infections (UTIs), bloodstream infections, and wound infections in human |
| *Yersinia enterocoletica* strain Y11 | Yep_Y11 | Yersiniaceae | Cause gastrointestinal infection in human |
