# Supplementary material for: Virulence Regulation and Lifestyle Transitions: The Role of c‐di‐GMP and Two‐Component Systems in Erwinia amylovora and Their Evolutionary Context Within Enterobacterales
Source: Mol Plant Pathol. 2026 Feb 16;27(2):e70228. doi: 10.1111/mpp.70228 (PMC12910134; doi:10.1111/mpp.70228)
Supplement: Supplementary file 6 — Table S2: Comparative distribution of c‐di‐GMP turnover domains (GGDEF, and EAL) across species within the Enterobacterales order. [file MPP-27-e70228-s002.docx]

**Supplementary Table S2:** Comparative distribution of c-di-GMP turnover domains (GGDEF, and EAL) across species within the Enterobacterales order.

| Species | Acronyms | GGDEF domain | EAL domain | Both GGDEF & EAL domain |
| --- | --- | --- | --- | --- |
|  |  | No. of Proteins | No. of Proteins | No. of Proteins |
| *Citrobacter ferundii* MSB1 1H | Cf_MSB1_1H | 11 | 13 | 8 |
| *Dickeya dianthicola* strain ME23 | Dd_ME23 | 8 | 3 | 2 |
| *Dickeya solani* strain IPO2222 | Ds_IPO2222 | 11 | 5 | 2 |
| *Escherichia coli* strain K12 | Ec_K12 | 12 | 10 | 7 |
| *Enterobacter cochlae* strain FDAARGOS 1431 | E_clo_FDAARGOS1431 | 14 | 16 | 8 |
| *Erwinia amylovora* strain Ea1189 | Ea_1189 | 4 | 4 | 4 |
| *Erwinia amylovora* strain ATCC15580 | Ea_ ATCC15580 | 4 | 4 | 4 |
| *Klebsiella pneumoniae* strain D23 | Kp_D23 | 13 | 20 | 5 |
| *Pantoea annatis* strain PA13 | Pa_PA13 | 10 | 10 | 8 |
| *Pectobacterium brasiliense* strain Pb1692 | Pb_1692 | 13 | 8 | 3 |
| *Pectobacterium carotovorum* strain WPP14 | Pc_WPP14 | 15 | 7 | 4 |
| *Salmonella enterica* serovar Typhi strain Ty2 | St_Ty2 | 5 | 9 | 6 |
| *Yersinia enterocoletica* strain Y11 | Yep_Y11 | 10 | 7 | 4 |
